# Influenza A Virus Uses PSMA2 for Downregulation of the NRF2-Mediated Oxidative Stress Response

**DOI:** 10.1128/jvi.01990-21

**Published:** 2022-03-09

**Authors:** Mahamud-ur Rashid, Ang Gao, Kevin M. Coombs

**Affiliations:** a Department of Medical Microbiology and Infectious Diseases, University of Manitoba, Winnipeg, Manitoba, Canada; b Manitoba Centre for Proteomics and Systems Biology, Winnipeg, Manitoba, Canada; c Children’s Hospital Research Institute of Manitoba, Winnipeg, Manitoba, Canada; Instituto de Biotecnologia/UNAM

**Keywords:** PSMA2, proteasome, NRF-2, oxidative stress, influenza A virus, host protein

## Abstract

Influenza A virus (IAV), an obligatory intracellular parasite, uses host cellular molecules to complete its replication cycle and suppress immune responses. Proteasome subunit alpha type 2 (PSMA2) is a cellular protein highly expressed in IAV-infected human lung epithelial A549 cells. PSMA2 is part of the 20S proteasome complex that degrades or recycles defective proteins and involves proteolytic modification of many cellular regulatory proteins. However, the role of PSMA2 in IAV replication is not well understood. In this study, PSMA2 knockdown (KD) in A549 cells caused a significant reduction in extracellular progeny IAV, but intracellular viral protein translation and viral RNA transcription were not affected. This indicates that PSMA2 is a critical host factor for IAV maturation. To better understand the interplay between PSMA2 KD and IAV infection at the proteomic level, we used the SomaScan 1.3K version, which measures 1,307 proteins to analyze alterations induced by these treatments. We found seven cellular signaling pathways, including phospholipase C signaling, Pak signaling, and nuclear factor erythroid 2p45-related factor 2 (NRF2)-mediated oxidative stress response signaling, that were inhibited by IAV infection but significantly activated by PSMA2 KD. Further analysis of NRF2-mediated oxidative stress response signaling indicated IAV inhibits accumulation of reactive oxygen species (ROS), but ROS levels significantly increased during IAV infection in PSMA2 KD cells. However, IAV infection caused significantly higher NFR2 nuclear translocation that was inhibited in PSMA2 KD cells. This indicates that PSMA2 is required for NRF2-mediated ROS neutralization and that IAV uses PSMA2 to escape viral clearance via the NRF2-mediated cellular oxidative response.

**IMPORTANCE** Influenza A virus (IAV) remains one of the most significant infectious agents, responsible for 3 million to 5 million illnesses each year and more than 50 million deaths during the 20th century. The cellular processes that promote and inhibit IAV infection and pathogenesis remain only partially understood. PSMA2 is a critical component of the 20S proteasome and ubiquitin-proteasome system, which is important in the replication of numerous viruses. This study examined host protein responses to IAV infection alone, PSMA2 knockdown alone, and IAV infection in the presence of PSMA2 knockdown and determined that interfering with PSMA2 function affected IAV maturation. These results help us better understand the importance of PSMA2 in IAV replication and may pave the way for designing additional IAV antivirals targeting PSMA2 or the host proteasome for the treatment of seasonal flu.

## INTRODUCTION

Influenza A virus (IAV) remains one of the most significant pathogens in human history. IAV caused over 50 million deaths worldwide just in the last century (1, 2), and every year 3 million to 5 million people get infected with severe illness and approximately 500,000 die worldwide (3). IAV belongs to the family *Orthomyxoviridae* and is further classified into subtypes based on hemagglutinin (HA) and neuraminidase (NA) antigens. To date, there are 18 HA and 11 NA types that have been discovered (4, 5). Among them, H1N1 and H3N2 are the most common causes of seasonal flu (3).

IAV has a highly mutation-prone genome consisting of 8 segments of negative-sense single-stranded RNA (4). The genomic plasticity enables the virus to evolve rapidly and become resistant to antiviral drugs and vaccines. Therefore, designing an effective vaccine has proven difficult and treatment has become more challenging (6). As viruses are intracellular parasites, they use the host cell machinery to facilitate replication and escape the immune response during infection. Hence, virus replication could be potentially prevented inside the host cell by blocking protein function or signaling pathways necessary for viral replication. Thus, it is important to clearly understand the IAV infection mechanism within host cells.

Virus-induced changes are reflected by dysregulation of host cellular proteins involved in different cellular pathways (7–9). Previous proteomic studies from our lab (7, 10, 11) and others (12, 13) found hundreds of dysregulated host proteins. Many of these interact with fibronectin (FN-1) (7, 14, 15). FN-1 is an extracellular matrix glycoprotein required for differentiation, migration, and cell adhesion (16, 17). FN-1 was found to be important for hepatitis B virus, gammaretrovirus, and rhabdovirus entry into host cells (18–20). In addition, some FN-1-interacting proteins were also reported to facilitate the entry of Ebola virus and human parvovirus B19 (21, 22).

IAV also uses FN-1 for virus attachment via 2,6-sialic acid binding and entry into human lung epithelial cells (23). However, different pathogenic strains of IAV were found to significantly affect expression of FN-1-interacting proteins. One such protein is PSMA2, whose levels were higher in highly pathogenic IAV-infected cells (7). Higher protein levels could result from protein upregulation, slower protein turnover, or a combination of both but for simplicity are referred to here as “upregulated expression.” Conversely, levels of proteins that are lower, because of either downregulation, faster turnover, or both, are referred to as “downregulated expression.” Additionally, IAV infection enhanced the activity profile of PSMA2 in lung epithelial cells (24).

PSMA2 is one of the seven alpha subunits in the 20S proteasome that form a ring structure that serves as a substrate entrance gate (25, 26). PSMA2 is critical for genome replication of West Nile virus (27). The proteosomes work in concert with ubiquitin in the ubiquitin-proteasome system (UPS). This complex is mainly involved in unfolding and degrading defective proteins in cells and thus regulates different cellular processes such as cell growth and differentiation, gene transcription, posttranslational modifications, and oxidative stress responses (28–31). The UPS facilitates the entry of murine coronavirus (32) and mouse minute virus (33, 34) and the release of HIV from the plasma membrane (35–37). Proteasome inhibition adversely impacts viral RNA and protein synthesis and reduces the replication of coxsackievirus (38).

So far, no study we are aware of has investigated the importance of PSMA2 in the influenza virus replication cycle. Therefore, we explored the role of this protein in IAV replication steps and the cellular signaling pathways during human lung cell infection. This study will help us understand the importance of PSMA2 in IAV replication and may provide us with novel therapeutic possibilities for combating influenza virus infection in the future.

## RESULTS

### Identification of IAV host dependency factors by siRNA screening.

**FIG 1:**
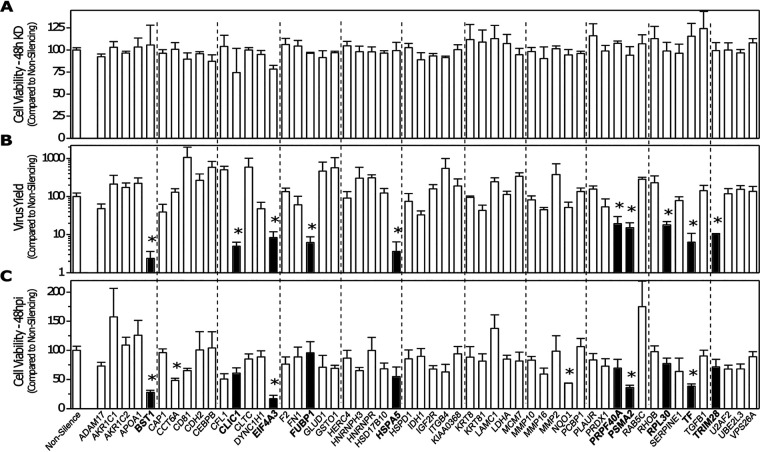
siRNA array screen of selected fibronectin-interacting proteins shown previously to be up- or downregulated by IAV infection. (A) Viability of cells transfected with a 100 nM concentration of the indicated siRNA was determined at 48 h posttransfection (hpt) by WST-1 assay. (B) Indicated 48-h-knockdown cells were infected with PR8 at an MOI of 0.02, supernatants were harvested at 42 hpi, and viral progeny replication was determined by plaque assay on MDCK cells. (C) Viability of the 48-h-transfected/48-h-infected cells determined by WST-1 assay. All assays performed in triplicate, with average values compared to the cognate values of cells transfected with a scrambled siRNA control (nonsilencing). Error bars represent SEM, and values determined to be statistically significantly altered (*P* < 0.05) are indicated with an asterisk.

A previous study from our lab by Simon and colleagues (7) showed that high- and low-pathogenicity IAV strains caused dysregulation of many A549 human lung FN-1-interacting cellular proteins. To understand the role of these FN-1-interacting proteins, a high-throughput, 96-well-based custom small interfering RNA (siRNA) screening was performed targeting 56 proteins. The impact of siRNA treatment on A549 cell viability and IAV replication was determined by WST-1 and plaque assay, respectively (Fig. 1). Knockdown (KD) of most of these genes had a minimal effect upon cell viability 48 h after transfection. Virus titer also was not affected by KD of most of the genes by 48 h postinfection (hpi). However, KD of BST1, CLIC1, EIF4A3, FUBP1, HSPA5, PRPF40A, PSMA2, RPL30, TF, and TRIM28 resulted in a significant (>3-fold) reduction in virus titers, whereas KD of CD81, CEBPB, CFL1, CLTC, GLUD1, GSTO1, ITGB4, MCM7, and MMP2 resulted in >3-fold enhancement of virus replication.

### Impact of PSMA2 KD on IAV replication.

**FIG 2:**
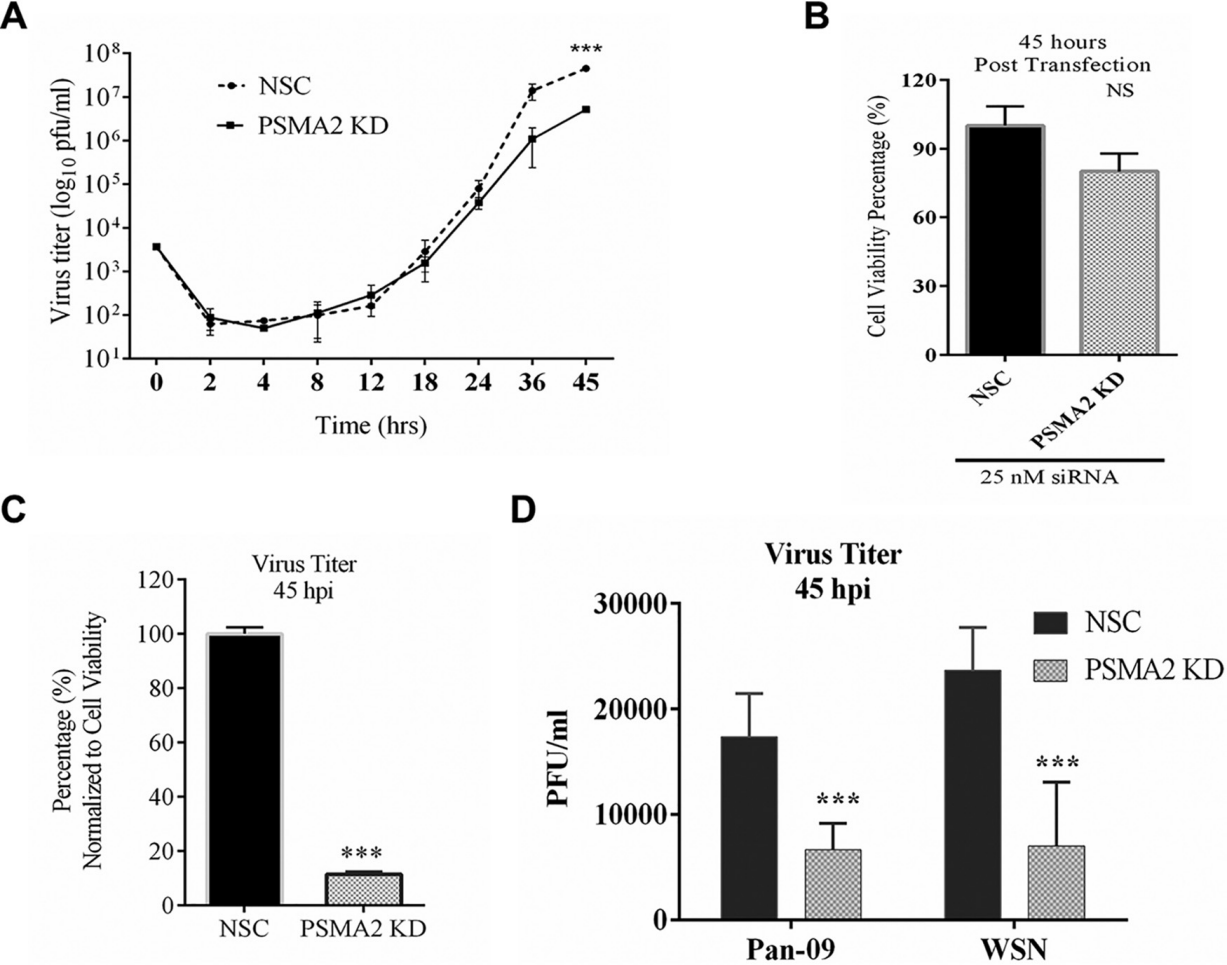
PSMA2 is required for replication of IAV. A549 cells were treated with either nonsilencing siRNA (NSC) or PSMA2 siRNA (PSMA2 knockdown [KD]) for 48 h and infected with IAV PR8 at an MOI of 0.01. Supernatants from the infected cells were collected at 0, 2, 4, 8, 12, 18, 24, 36, and 45 h postinfection (hpi). Similarly, NSC and PSMA2 KD cells were infected with IAV strains pdm09 and WSN and supernatants were collected at 45 hpi. Virus titers were determined by plaque assay. (A) Influenza A virus (PR8 strain) titer in the PSMA2 KD cell supernatant compared to the control (NSC) over time. (B) Viability of cells measured by WST-1 assay at 45 h post-siRNA transfection. (C) Percentage of virus titer in PSAM2 KD cell supernatant at 45 hpi compared to the control and normalized with cell viability. (D) Impact of PSMA2 KD on IAV pdm09 and WSN strains. NS, not significant. ***, *P* < 0.001.

We focused on PSMA2 because of its key role in the proteasome and UPS, because little is known about its role in viral replication, and because of reagent availability. To understand the role of PSMA2 in the IAV replication cycle initially, we determined the impact of PSMA2 KD on progeny virus replication. A549 cells were infected with PR8 after PSMA2 KD. The supernatants were collected at different time points up to 45 hpi. The progeny virus titer was determined by plaque assay. PSMA2 KD caused a significant reduction of progeny viruses in the supernatant at 45 hpi (Fig. 2A). PSMA2 KD caused a reduction in cell viability that was not statistically significant (Fig. 2B). The virus titer in the supernatant was normalized to cell viability; this indicated about 90% reduction of virus titer from PSMA2 KD cells (Fig. 2C). The impact of PSMA2 KD was not restricted only to the PR8 strain but also caused a significant reduction in pdm09 and WSN virus replication (Fig. 1D). These data indicate that PSMA2 is required for the successful completion of IAV replication.

### Impact of PSMA2 KD on IAV translation, transcription, and intracellular localization.

**FIG 3:**
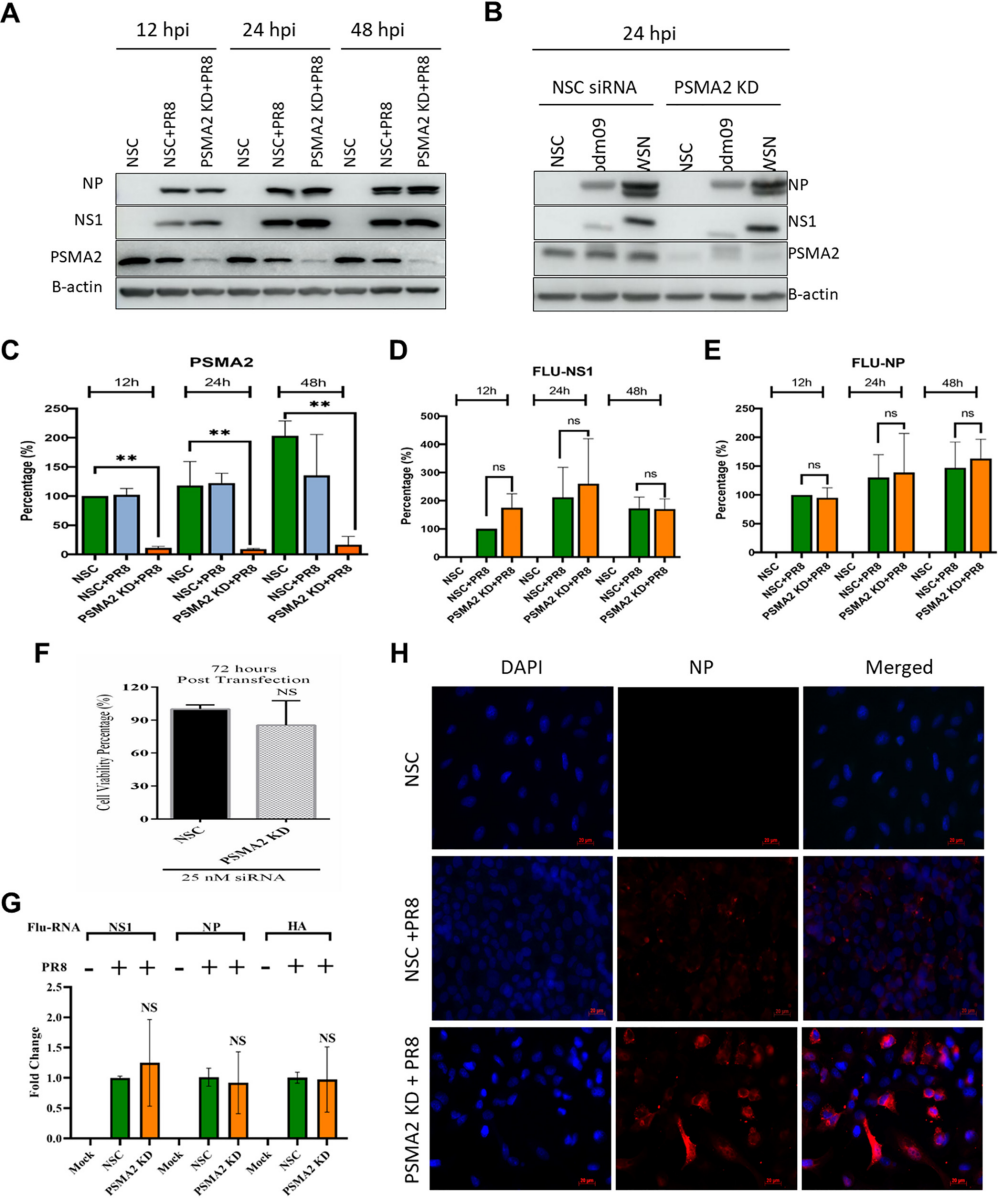
PSMA2 KD does not impact translation of viral proteins and transcription of vRNAs but impacts maturation. A549 cells were treated with either nonsilencing siRNA (NSC) or PSMA2 siRNA (PSMA2 KD) for 48 h and infected with IAV PR8, pdm09, or WSN at an MOI of 3. Cell lysates were collected at 12, 24, and 48 hpi from PR8-infected cells and at 24 hpi from pdm09-infected and WSN-infected cells for analyzing the expression of viral proteins by Western blotting. After 24 hpi, cells were fixed on slides to measure viral protein localization by immunofluorescence microscopy. Viral RNAs were collected at 24 hpi, and the comparative vRNA transcripts were determined by qRT-PCR. (A) Expression of IAV PR8 NP and NS1 proteins in PSMA2 cells at 12, 24, and 48 hpi. (B) Expression of viral proteins in PSMA2 KD at 24 hpi after infection with pdm09 and WSN strains. (C to E) Quantitative densitometry analysis of Western blot images to determine knockdown of PSMA2 expression (C), IAV NS1 protein expression (D), and Flu-NP protein expression (E). (F) Impact of PSMA2 KD on cell viability measured by WST-1 assay at 72 h after transfection by PSMA2 siRNA. (G) IAV NS1, NP, and HA vRNA transcripts in PSMA2 KD cells compared to mock-infected cells and NSC control. (H) Immunofluorescence images showing the expression of IAV NP protein in infected PSMA2 KD cells. *, *P* < 0.05; **, *P* < 0.01; ***, *P* < 0.001.

Since IAV progeny virus production was significantly reduced in PSMA2 KD cells, we investigated the specific step(s) in virus replication that was affected by PSMA2 KD. First, we assessed the impact on viral protein translation. PSMA2 KD, and scrambled nonsilencing siRNA (NSC), A549 cells were infected with PR8 at a multiplicity of infection (MOI) of 3. The infected cells were harvested at 12, 24, and 48 hpi. IAV NS1 and NP proteins were detected by Western blotting from cell lysates (Fig. 3A). Although PSMA2 KD caused a significant impact on progeny virus yield, it did not impact viral protein synthesis (Fig. 3C to E). As PR8 is a lab-adapted strain, we also tested PSMA2 KD effects on the translation of other human IAV strain proteins. As for PR8, we did not observe any significant differences in pdm09 or WSN viral protein translation (Fig. 3B). A549 cell viability was not significantly affected by PSMA2 KD even 72 h after transfection (Fig. 3F).

We next tested the impact of PSMA2 KD on the transcription of viral RNAs (vRNAs) by reverse transcription-quantitative PCR (qRT-PCR). PSMA2 KD and NSC cells were infected at an MOI of 3 and RNA was extracted from the cells at 24 hpi. cDNA was prepared by reverse transcription and qPCR was performed targeting NS1, NP, and HA vRNAs. As with protein translation, PSMA2 KD did not have a significant impact on any of the targeted vRNA transcription processes (Fig. 3G).

To further assess the impact of PSMA2 KD on the localization of viral proteins, PSMA2 KD and NSC cells were infected at an MOI of 3 and cells were fixed at 24 hpi. Immunofluorescence (IF) microscopy was performed, targeting IAV NP protein. We observed that NP protein intensity was higher in many PSMA2 KD cells than in control NSC-treated infected cells (Fig. 3H).

### Proteomic dysregulation caused by PSMA2 KD during IAV infection.

**FIG 4:**
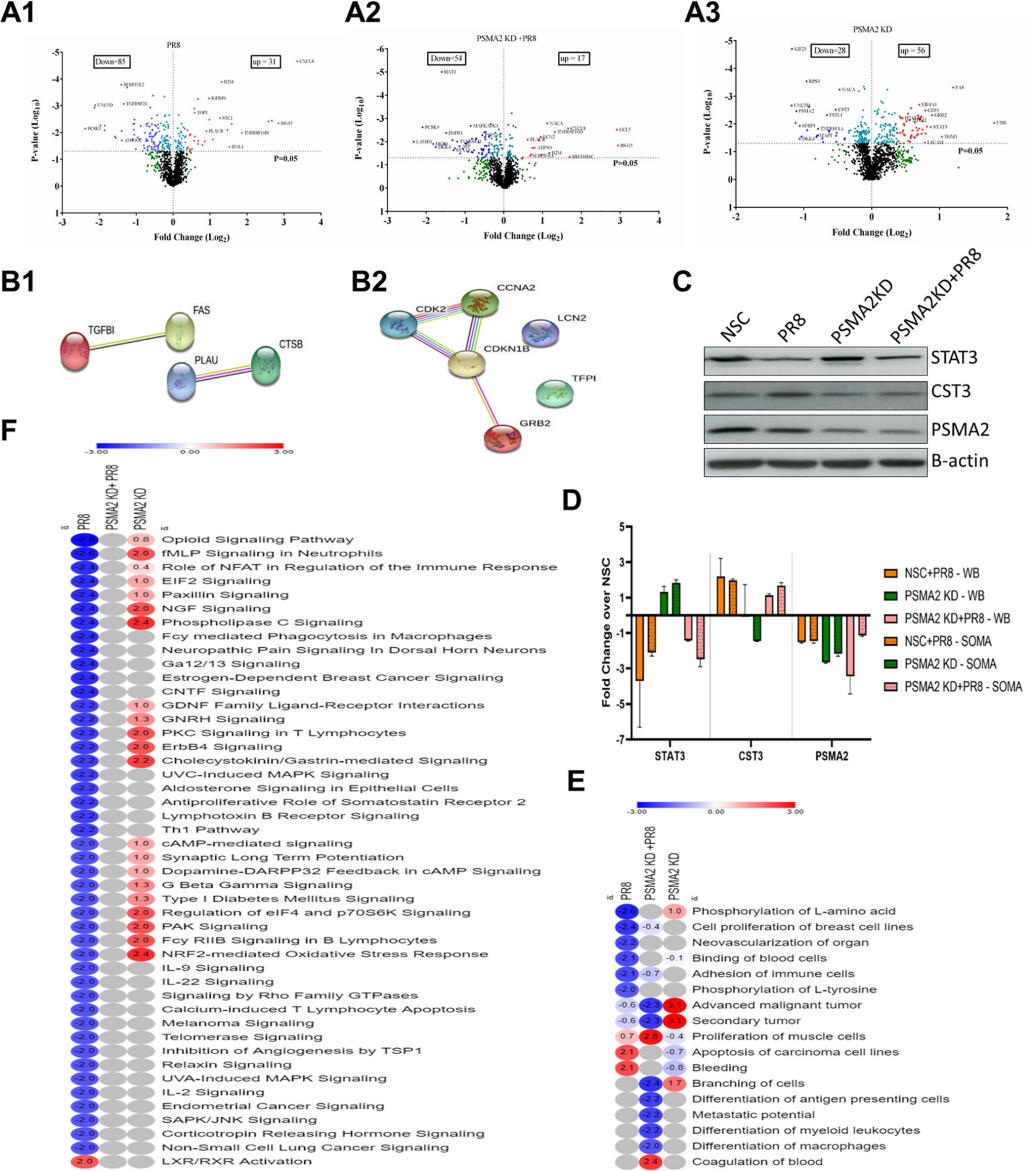
Proteomic analysis to delineate the impact of PSMA2 KD on IAV replication. NSC and PSMA2 KD cells were infected with IAV PR8 at an MOI of 3. Cell lysates were collected from uninfected NSC and PSMA2 KD cells and after infection with PR8 at 24 hpi. Uninfected NSC and PSMA2 KD cells were used as controls. Cell lysates were analyzed by the SomaScan platform, which can detect >1,300 predefined proteins simultaneously from each sample. The protein expression values were compared between the groups to determine whether the protein dysregulation was an experimental condition. PSMA2 KD versus NSC, NSC infected with PR8 versus NSC, and PSMA2 KD infected with PR8 versus PSMA2 KD comparisons were made to determine the impact of PSMA2 knockdown (PSMA2 KD), PR8 infection (PR8) and impact of PR8 infection in PSMA2 KD cells (PSMA2 KD+PR8), respectively. (A) Volcano plot of proteins dysregulated in IAV PR8-infected (A1), PSMA2 KD (A2), and IAV PR8-infected PSMA2 KD (A3) cells. (B1) Proteins significantly dysregulated by PR8 infection but with an opposite trend of expression in PSMA2 KD+PR8 cells. (B2) Proteins significantly dysregulated by PSMA2 KD+PR8 infection but with an opposite trend of expression in PR8-infected cells. (C) Validation SomaScan data by Western blot detection of STAT3, CST3, and PSMA2 proteins. (D) Quantitative densitometry of Western blot images and comparison with SomaScan data for data validation. (E) Heat map of the disease and functions significantly dysregulated by either PR8 or PSMA2 KD+PR8 but not by the others. (F) Heat map of significantly dysregulated canonical pathways in PR8-infected cells; significance could not be predicted by IPA in PSMA2 KD+PR8 cells.

To better understand the role of PSMA2 in the IAV replication cycle, we further evaluated the impact of PR8 in PSMA2 KD cells by measuring dysregulation of the cellular proteome. Cellular proteomic dysregulation was determined by the SomaScan platform, which can perform quantitative measurements of 1,307 proteins simultaneously from up to 92 samples (39). The impacts of PR8 infection alone, PSMA2 KD alone, and PR8 infection of PSMA2 KD cells (PSMA2 KD+PR8) were determined by comparing the cellular proteomes in PR8-infected versus NSC, PSMA2 KD versus NSC, and PSMA2 KD+PR8 versus PSMA2 KD cells alone, respectively. PSMA2 KD, PR8 infection and PSMA2 KD+PR8 infection caused significant dysregulation of 272, 218, and 149 proteins (Table 1), respectively. However, by employing cutoff values of ≥+1.5 or ≤−1.5-fold change and *P* values of <0.05, a total of 52 (32 upregulated and 20 downregulated) proteins from PSMA2 KD, 71 (21 upregulated and 50 downregulated) proteins from PR8 infection, and 46 (15 upregulated and 31 downregulated) proteins from PSMA2 KD+PR8 infection were selected for further bioinformatics analyses (Table 2). Volcano plot analyses of dysregulated proteins indicated that many proteins were differentially dysregulated between PR8-infected and PSMA2 KD+PR8-infected cells (Fig. 4A1, A2, and A3). Transforming growth factor beta-induced (TGFBI), Fas cell surface death receptor (FAS), plasminogen activator urokinase (PLAU), and cathepsin B (CTSB) proteins were significantly dysregulated by PR8 infection but had an opposite trend of expression in PSMA2 KD+PR8-infected cells (Fig. 4B1). Cyclin-dependent kinase 2 (CDK2), cyclin A2 (CCNA2), cyclin-dependent kinase inhibitor 1B (CDKN1B), lipocalin 2 (LCN2), tissue factor pathway inhibitor (TFPI), and growth factor receptor-bound protein 2 (GRB2) proteins were significantly dysregulated by PSMA2 KD+PR8 infection but had an opposite trend of expression in PR8-infected non-KD cells (Fig. 4B2). However, 68 proteins were significantly dysregulated by PR8 infection but not significantly dysregulated under the PSMA2 KD+PR8 condition, and 21 proteins were significantly dysregulated in PSMA2 KD+PR8-infected cells but were not significantly dysregulated by PR8 infection (see Fig. S1 in the supplemental material).

**TABLE 1:** Numbers of host proteins significantly dysregulated by PSMA2 KD alone, PR8 infection alone, and PR8 infection in PSMA2 KD cells^*a*^

Range of fold change	No. of proteins					
	PSMA2 KD	Total significant	PR8	Total significant	PSMA2 KD +PR8	Total significant
and F.C. >1.00	140	272	76	218	50	149
and F.C. <1.00	132		142		99	
and F.C. >1.10	121	202	68	197	36	121
and F.C. <−1.10	81		129		85	
and F.C. >1.20	88	120	43	144	19	86
and F.C. <−1.20	32		101		67	
and F.C. >1.30	56	84	31	116	17	71
and F.C. <−1.30	28		85		54	
**and F.C. >1.50**	**32**	**52**	**21**	**71**	**15**	**46**
**and F.C. <−1.50**	**20**		**50**		**31**	
and F.C. >1.60	22	39	17	57	13	42
and F.C. <−1.60	17		40		29	
and F.C. >2.00	3	10	11	33	7	42
and F.C. <−2.00	7		22		35	
and F.C. >2.50	1	1	8	22	4	13
and F.C. <−2.50	0		14		9	

a Significance was determined by *t*-test and Z-score as detailed in Materials and Methods from three biological replicates. The complete list of proteins dysregulated ≥1.5-fold in either direction is provided in Table 2. Bold represents those proteins presented in Table 2.

**TABLE 2:** Host proteins significantly dysregulated by PSMA2 KD alone, PR8 infection alone, and PR8 infection in PSMA2 KD cells^*a*^

Type of protein	Gene	Entrez gene name	PSMA2 KD (FC)	*P* value	PR8 (FC)	*P* value	PSMA2 KD+PR8 (FC)	*P* value	Location
Cytokines	CXCL8	C-X-C motif chemokine ligand 8	−1.1	0.009	**10.3**	2.2E−05	**3.2**	0.003	Extracellular space
	IFNL1	Interferon lambda 1	1.1	0.24	**2.9**	0.035	**1.9**	0.037	Extracellular space
	CCL13	C-C motif chemokine ligand 13	−1	0.86	−1.4	0.094	**−1.6**	0.031	Extracellular space
	CCL5	C-C motif chemokine ligand 5	1	0.87	**6.4**	0.004	**7.5**	0.003	Extracellular space
Enzymes	UBB	Ubiquitin B	**3.5**	0.009	1.1	0.54	−1.4	0.12	Cytoplasm
	TGM3	Transglutaminase 3	**2**	0.029	−1.1	0.84	−1.4	0.14	Cytoplasm
	UFC1	Ubiquitin fold modifier conjugating enzyme 1	**1.6**	0.007	1.2	0.31	1	0.40	Cytoplasm
	EIF4A3	Eukaryotic translation initiation factor 4A3	**1.6**	0.002	1.1	0.16	1	0.84	Nucleus
	TYMS	Thymidylate synthetase	**1.6**	0.017	−1.1	0.12	**−1.8**	0.028	Nucleus
	AKR1A1	Aldo-keto reductase family 1 member A1	1.2	0.043	**−1.7**	0.038	−1.1	0.56	Cytoplasm
	RPS3	Ribosomal protein S3	**−1.9**	2.9E−04	1.3	0.69	1.4	0.16	Cytoplasm
	PPIF	Peptidylprolyl isomerase F	1.3	0.086	**1.7**	0.029	1.3	0.06	Cytoplasm
	PPID	Peptidylprolyl isomerase D	1.4	0.14	**−1.5**	0.010	−1.4	0.33	Cytoplasm
	CNTN1	Contactin 1	−1.1	0.35	**−2.9**	0.016	**−2.9**	0.013	Plasma membrane
	HAT1	Histone acetyltransferase 1	2.4	0.37	**−2.4**	2.1E−04	**−3**	1.0E−05	Nucleus
	TOP1	DNA topoisomerase I	−1	0.95	**1.5**	0.002	1.6	0.34	Nucleus
Growth factors	DKK1	Dickkopf WNT signaling pathway inhibitor 1	**−2**	0.029	**−3.6**	0.006	**−3.5**	0.013	Extracellular space
	BMP6	Bone morphogenetic protein 6	1.2	0.062	**−1.6**	0.032	−1.4	0.09	Extracellular space
	FGF6	Fibroblast growth factor 6	−1.2	0.22	**−1.6**	0.006	−1.4	0.098	Extracellular space
	NRG1	Neuregulin 1	1.1	0.81	**−1.6**	0.047	−1.6	0.40	Plasma membrane
	GRN	Granulin precursor	1	0.98	**−2.1**	0.005	−1.6	0.069	Extracellular space
Kinases	PDPK1	3-Phosphoinositide-dependent protein kinase 1	**1.7**	0.022	−1.5	0.061	−1.5	0.18	Cytoplasm
	AK1	Adenylate kinase 1	**1.7**	0.016	−1.1	0.58	−1	0.63	Cytoplasm
	MAP2K3	Mitogen-activated protein kinase 3	**1.7**	0.008	1.3	0.043	1.1	0.50	Cytoplasm
	SPHK1	Sphingosine kinase 1	**1.6**	0.007	1.3	0.11	−1.1	0.55	Cytoplasm
	EPHA2	EPH receptor A2	**1.6**	0.015	**3**	0.005	1.7	0.11	Plasma membrane
	CSNK2A2	Casein kinase 2 alpha 2	**1.6**	0.006	−1.4	0.086	**−1.7**	0.029	Cytoplasm
	CSNK2B	Casein kinase 2 beta	**1.6**	0.006	−1.4	0.086	**−1.7**	0.029	Cytoplasm
	MAPK8	Mitogen-activated protein kinase 8	**1.6**	0.014	−1.2	0.35	−1.4	0.25	Cytoplasm
	WNK3	WNK lysine-deficient protein kinase 3	**1.6**	0.009	−1.1	0.32	−1.1	0.40	Plasma membrane
	PRKCI	Protein kinase C iota	**1.5**	0.017	1.4	0.097	1.2	0.12	Cytoplasm
	NAGK	*N*-Acetylglucosamine kinase	1.3	0.04	**−1.5**	0.035	−1.2	0.28	Cytoplasm
	PRKACA	Protein kinase cAMP-activated catalytic subunit alpha	**−1.5**	0.017	−1.2	0.38	−1.1	0.39	Cytoplasm
	CDK2	Cyclin-dependent kinase 2	**−1.9**	0.017	**−1.3**	0.22	1.4	0.048	Nucleus
	EFNA2	Ephrin A2	−1.4	0.055	**−1.7**	0.005	−1.4	0.099	Plasma membrane
	STC1	Stanniocalcin 1	−1.3	0.057	**2.4**	0.003	**1.6**	0.036	Extracellular space
	MAPKAPK2	MAPK-activated protein kinase 2	1.2	0.08	−1.5	0.014	**−1.6**	0.045	Nucleus
	PIK3CA	Phosphatidylinositol-4,5-bisphosphate 3-kinase catalytic subunit alpha	1.1	0.19	**−1.6**	0.043	−1.5	0.14	Cytoplasm
	PIK3R1	Phosphoinositide-3-kinase regulatory subunit 1	1.1	0.19	**−1.6**	0.043	−1.5	0.14	Cytoplasm
	MET	MET proto-oncogene, receptor tyrosine kinase	−1	0.26	−1.5	0.094	**−2.1**	0.020	Plasma membrane
	CAMK2D	Calcium/calmodulin-dependent protein kinase II delta	1.3	0.32	**−1.5**	0.042	−1.4	0.18	Cytoplasm
	CAMK2B	Calcium/calmodulin-dependent protein kinase II beta	1.2	0.44	**−1.6**	0.006	−1.5	0.23	Cytoplasm
	EPHA3	EPH receptor A3	1	0.44	**1.6**	0.020	1.2	0.16	Plasma membrane
	FGFR1	Fibroblast growth factor receptor 1	−1	0.45	**−2.1**	0.003	**−2.8**	0.004	Plasma membrane
	PRKCG	Protein kinase C gamma	1	0.63	**−1.6**	0.020	−1.4	0.048	Cytoplasm
	MAPKAPK3	MAPK-activated protein kinase 3	−1	0.74	−1.7	0.061	**−1.8**	0.002	Nucleus
Peptidases	PSMA1	Proteasome 20S subunit alpha 1	**1.6**	0.023	−1.2	0.066	−1.9	0.064	Cytoplasm
	IDE	Insulin-degrading enzyme	**1.6**	0.026	1.1	0.48	1.5	0.48	Extracellular space
	CTSA	Cathepsin A	−1.4	0.044	**−2.1**	0.005	**−1.7**	0.024	Cytoplasm
	CTSV	Cathepsin V	**−1.5**	0.019	−1.1	0.33	1	0.93	Cytoplasm
	PSMA2	Proteasome 20S subunit alpha 2	**−2.2**	0.004	−1.4	0.024	−1.1	0.15	Cytoplasm
	PCSK9	Proprotein convertase subtilisin/kexin type 9	**−2.2**	0.009	**−5.2**	0.007	**−4.3**	0.002	Extracellular space
	C1R	Complement C1r	1	0.64	1.5	0.20	**1.9**	0.009	Extracellular space
Phosphatase	PPP3CA	Protein phosphatase 3 catalytic subunit alpha	**1.5**	0.025	−1.3	0.17	−1.2	0.30	Cytoplasm
	PPP3R1	Protein phosphatase 3 regulatory subunit B alpha	**1.5**	0.025	−1.3	0.17	−1.2	0.30	Cytoplasm
	PTPN6	Protein tyrosine phosphatase non-receptor type 6	−1	0.40	**−1.5**	0.001	−1.5	0.005	Cytoplasm
Transcription regulators	STAT3	Signal transducer and activator of transcription 3	**1.8**	0.013	**−2.1**	0.008	**−2.5**	0.023	Nucleus
	ARID3A	AT-rich interaction domain 3A	**1.8**	0.012	−1.1	0.46	−1.3	0.032	Nucleus
	TBP	TATA-box binding protein	**1.7**	0.005	−1.5	0.058	**−1.6**	0.007	Nucleus
	STAT6	Signal transducer and activator of transcription 6	1.2	0.042	**−1.7**	0.021	−1.4	0.015	Nucleus
	NACA	Nascent polypeptide-associated complex subunit alpha	−1.4	0.001	**2.1**	0.018	**2.2**	0.002	Cytoplasm
	EEF1B2	Eukaryotic translation elongation factor 1 beta 2	−1.7	0.034	1.1	0.49	1.4	0.054	Cytoplasm
	SMAD2	SMAD family member 2	1	0.20	**−1.6**	0.035	**−1.6**	0.008	Nucleus
	AIP	Aryl hydrocarbon receptor-interacting protein	1.1	0.78	**−1.6**	0.011	−1.2	0.54	Nucleus
	HMGB1	High-mobility-group box 1	1	0.89	**−1.5**	0.014	−1.2	0.16	Nucleus
Transmembrane receptors	FAS	Fas cell surface death receptor	**2.3**	4.8E−04	1.3	0.023	−1	0.96	Plasma membrane
	ITGB1	Integrin subunit beta 1	**1.6**	0.031	1.1	0.62	1.1	0.28	Plasma membrane
	TNFRSF21	TNF receptor superfamily member 21	−1.4	0.027	**−2.5**	0.001	**−2.3**	0.010	Plasma membrane
	GFRA1	GDNF family receptor alpha 1	**−1.6**	0.045	**−1.9**	0.015	−1.6	0.055	Plasma membrane
	TNFRSF1A	TNF receptor superfamily member 1A	**−1.7**	0.015	**−3.9**	0.005	**−2.5**	0.009	Plasma membrane
	SFRP1	Secreted frizzled-related protein 1	**−2.1**	0.012	−1.2	0.066	−1.3	0.22	Plasma membrane
	B2M	Beta 2 microglobulin	1.1	0.063	**2.5**	1.3E−04	**2.3**	0.031	Plasma membrane
	PLAUR	Plasminogen activator, urokinase receptor	−1	0.10	**1.9**	0.009	**1.5**	0.008	Plasma membrane
	RTN4R	Reticulon 4 receptor	−1.8	0.18	**−3**	0.014	**−1.6**	0.23	Plasma membrane
	TNFRSF10D	TNF receptor superfamily member 10d	−1.1	0.18	**3.6**	0.010	**2.5**	0.004	Plasma membrane
	KIR2DL4	Killer cell immunoglobulin-like receptor, two Ig domains, and long cytoplasmic tail 4	−1.1	0.22	**−1.7**	0.010	**−1.5**	0.035	Plasma membrane
	MICB	MHC class I polypeptide-related sequence B	−1.2	0.25	**−1.7**	0.030	**−1.9**	0.027	Plasma membrane
	PLXNB2	Plexin B2	1.1	0.38	−1.3	0.038	**−2**	0.012	Plasma membrane
	NRP1	Neuropilin 1	1	0.96	**−2.2**	0.018	**−2.4**	0.019	Plasma membrane
Transporters	BPI	Bactericidal permeability-increasing protein	−1.2	0.11	**−1.6**	0.005	−1.4	0.024	Plasma membrane
	SNX4	Sorting nexin 4	−1.1	0.35	**−1.6**	0.020	−1.4	0.18	Cytoplasm
	ATP5PO	ATP synthase peripheral stalk subunit OSCP	−1.1	0.45	**1.6**	0.027	**1.7**	0.018	Cytoplasm
	LCN2	Lipocalin 2	−1	0.68	−1.2	0.12	**1.9**	0.006	Extracellular space
Others	GRB2	Growth factor receptor-bound protein 2	**1.9**	0.005	1	0.93	**−1.6**	0.011	Cytoplasm
	CD55	CD55 molecule (Cromer blood group)	**1.7**	0.003	**1.6**	0.001	1.2	0.23	Plasma membrane
	L1CAM	L1 cell adhesion molecule	**1.7**	0.047	1.3	0.50	1.1	0.31	Plasma membrane
	NSFL1C	NSFL1 cofactor	**1.7**	0.022	−1.1	0.39	−1.3	0.077	Cytoplasm
	SBDS	SBDS ribosome maturation factor	**1.6**	0.006	1.1	0.70	−1.3	0.28	Nucleus
	ISG15	ISG15 ubiquitin like modifier	**1.6**	0.005	**7.2**	0.005	**7.7**	0.015	Extracellular space
	ITGA1	Integrin subunit alpha 1	**1.6**	0.031	1.1	0.62	1.1	0.28	Plasma membrane
	SHC1	SHC adaptor protein 1	**1.5**	0.029	−1.2	0.085	−1.2	0.42	Cytoplasm
	H2AZ1	H2A.Z variant histone 1	1.4	0.006	**−2.1**	0.012	**−1.9**	0.011	Nucleus
	CST3	Cystatin C	−1.4	0.003	**2**	0.003	**1.7**	0.019	Extracellular space
	FSTL1	Follistatin-like 1	**−1.6**	0.005	1.1	0.21	1.1	0.26	Extracellular space
	APP	Amyloid beta precursor protein	**−1.7**	0.026	**−1.9**	0.018	**−2.2**	0.016	Plasma membrane
	MAPT	Microtubule-associated protein tau	**−1.7**	0.026	−1.4	0.15	1.1	0.72	Plasma membrane
	AMIGO2	Adhesion molecule with Ig-like domain 2	**−1.8**	0.030	**−2.6**	0.019	**−1.9**	0.012	Plasma membrane
	SERPINE2	Serpin family E member 2	**−1.9**	0.002	**−2.6**	1.7E−04	**−1.8**	0.007	Extracellular space
	CCNA2	Cyclin A2	**−1.9**	0.017	−1.3	0.22	1.4	0.048	Nucleus
	DKK4	Dickkopf WNT signaling pathway inhibitor 4	**−2.1**	0.034	**−3.6**	0.008	**−3.4**	0.017	Extracellular space
	KIF23	Kinesin family member 23	**−2.3**	2.0E−05	**−2.6**	0.009	−1.3	0.47	Cytoplasm
	UNC5D	Unc-5 netrin receptor D	**−2.3**	0.002	**−4.3**	0.001	**−2.2**	0.032	Plasma membrane
	TGFBI	Transforming growth factor beta induced	−1.3	0.094	**1.5**	0.014	−1.2	0.19	Extracellular space
	IGFBP2	Insulin-like growth factor binding protein 2	−1.1	0.15	**1.8**	0.021	1.2	0.10	Extracellular space
	MICA	MHC class I polypeptide-related sequence A	−1.5	0.20	**−1.8**	0.049	−1.7	0.15	Plasma membrane
	RSPO2	R-spondin 2	−1.2	0.21	**−1.7**	0.018	−1.5	0.051	Extracellular space
	H1-2	H1.2 linker histone, cluster member	1.4	0.21	**2.8**	0.008	**3.2**	0.045	Nucleus
	GREM1	Gremlin 1, DAN family BMP antagonist	−1.2	0.22	**−1.5**	0.011	−1.4	0.064	Extracellular space
	CFH	Complement factor H	−1.2	0.24	**−1.8**	0.006	−1.4	0.13	Extracellular space
	LAMA1	Laminin subunit alpha 1	1.2	0.36	**−4.4**	0.001	**−5**	0.010	Extracellular space
	LAMB1	Laminin subunit beta 1	1.2	0.36	**−4.4**	0.001	**−5**	0.010	Extracellular space
	LAMC1	Laminin subunit gamma 1	1.2	0.36	**−4.4**	0.001	**−5**	0.010	Extracellular space
	SERPINE1	Serpin family E member 1	1.1	0.38	**6**	0.004	**1.6**	0.041	Extracellular space
	IGFBP6	Insulin-like growth factor binding protein 6	1.1	0.50	**2**	0.001	1.1	0.57	Extracellular space
	SLITRK5	SLIT and NTRK-like family member 5	1	0.59	**−1.6**	0.014	−1.5	0.057	Plasma membrane
	MFGE8	Milk fat globule EGF and factor V/VIII domain containing	−1	0.89	**−2**	0.042	−1.6	0.088	Extracellular space

a Significantly dysregulated fold changes (FC) (≥1.5 or ≤−1.5) are indicated in bold type. MAPK, mitogen-activated protein kinase; MHC, major histocompatibility complex.

Western blotting was performed targeting STAT3, CST3, and PSMA2 proteins to validate the SomaScan data (Fig. 4C). Quantitative densitometry of Western blot images showed that all three proteins followed the same expression trends as determined by SomaScan (Fig. 4D).

### Bioinformatics analysis of dysregulated proteins.

Ingenuity Pathway Analysis (IPA) was used to analyze the list of dysregulated proteins to understand the impact on diseases, functions, and cellular signaling pathways. IPA predicted that a total of 79 diseases and functions were dysregulated by at least one of the conditions (PR8, 3 upregulated and 18 downregulated; PSMA2 KD, 38 upregulated and 7 downregulated; and PSMA2 KD+PR8, 5 upregulated and 22 downregulated) (Table S1). Among them, 17 diseases and functions were significantly dysregulated by either PR8 infection alone or PSMA2 KD+PR8 infection but not by others. In this list, we found that l-tyrosine and l-amino acid phosphorylation were not affected during IAV infection in PSMA2 KD cells, but l-tyrosine and l-amino acid phosphorylation were downregulated in PR8-infected non-KD cells (Fig. 4E).

**FIG 5:**
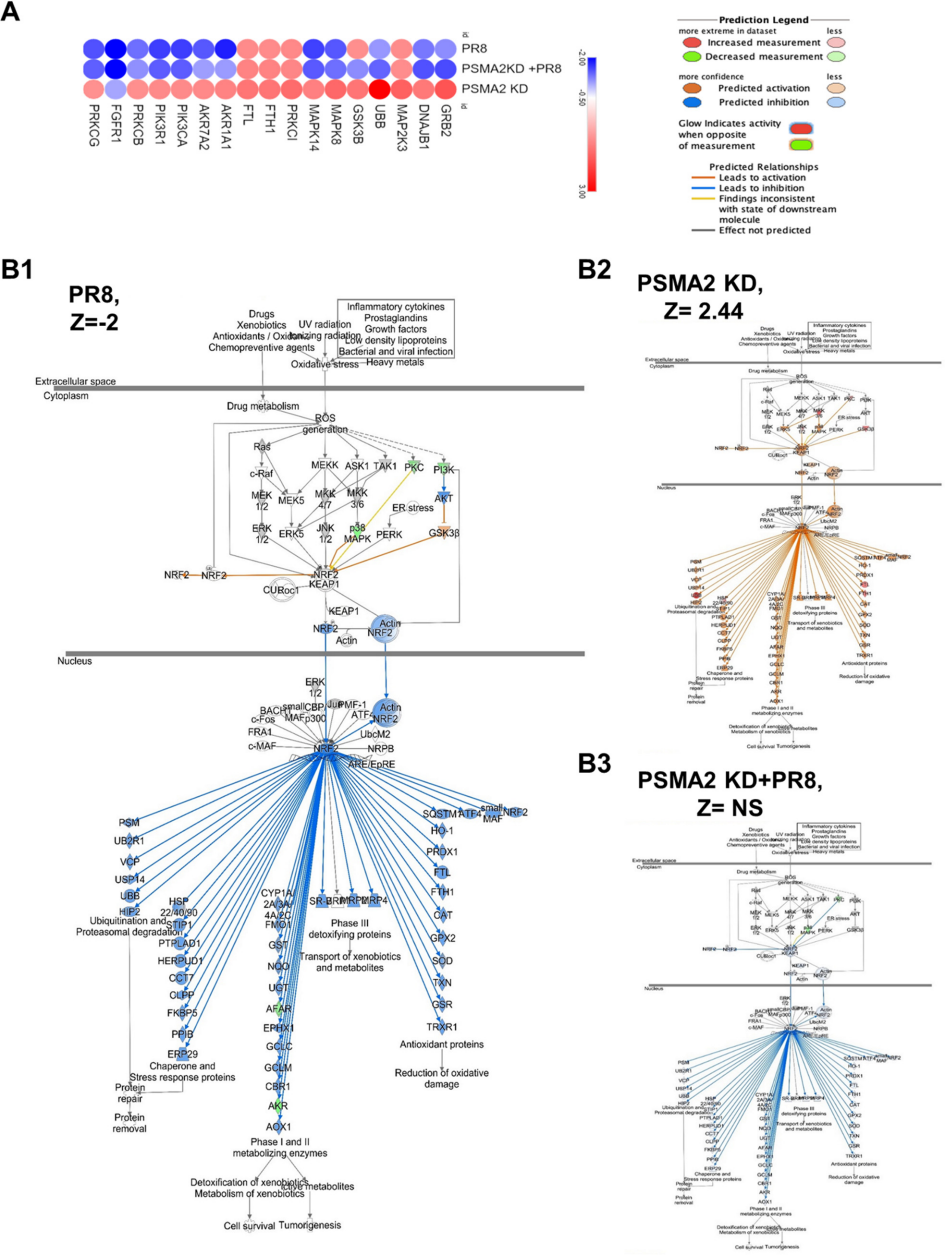
Influenza A virus uses PSMA2 for downregulation of NRF2-mediated oxidative stress response. (A) Proteins associated with NRF2-mediated oxidative stress response pathway dysregulated by PR8 infection, PSMA2 KD, and PSMA2 KD+PR8. Red, upregulated; blue, downregulated. (B) NRF2-mediated oxidative stress response signaling pathway activation by PR8 infection (B1), PSMA2 KD (B2), and PR8 infection with PSMA2 KD (B3). Orange and blue indicate IPA-predicted activation and inactivation, respectively. mpi, minutes postinfection.

However, IPA also predicted that PR8 infection can cause dysregulation of 121 canonical pathways (2 upregulated and 119 downregulated). In comparison, PSMA2 KD caused upregulation of 35 pathways and downregulation of only 1. Interestingly, IPA could predict that 61 (1 upregulated and 60 downregulated) pathways were significantly reregulated by PSMA2 KD+PR8 infection (Table S2). However, we found that 46 canonical pathways were significantly downregulated by PR8 infection but were not significantly affected by PSMA2 KD+PR8 (Fig. 4F). Among them, 10 pathways were activated considerably in PSMA2 KD cells; there was no impact in PSMA2 KD+PR8 infection by IPA prediction. Three of these pathways may not be relevant for lung epithelial cells, as those are immune cell specific. The remaining seven pathways are phospholipase C signaling, NGF signaling, ErbB4 signaling, PAK signaling, regulation of eIF4 and P7S6K signaling, cholecystokinin/gastrin-mediated signaling, and the nuclear factor erythroid 2p45-related factor 2 (NRF2)-mediated oxidative stress response (Fig. 4F). Based on relevance and the highest number of significantly dysregulated proteins in the pathway, we selected NRF2-mediated oxidative stress response signaling for further investigation (Fig. 5A).

### PSMA2 KD reduces the impact of IAV infection on NRF2-mediated oxidative stress response signaling.

Based on the dysregulated proteins, IPA predicted that PR8 infection could cause significant downregulation of the NRF2-mediated signaling pathway (Z-score = −2 [Fig. 5B1]) but a significant upregulation of this pathway by PSMA2 KD (Z-score = +2.44 [Fig. 5B2]). However, IPA could not predict any significant impact on this pathway during IAV infection of PSMA2 KD cells (Z-score = not significant [Fig. 5B3]).

**FIG 6:**
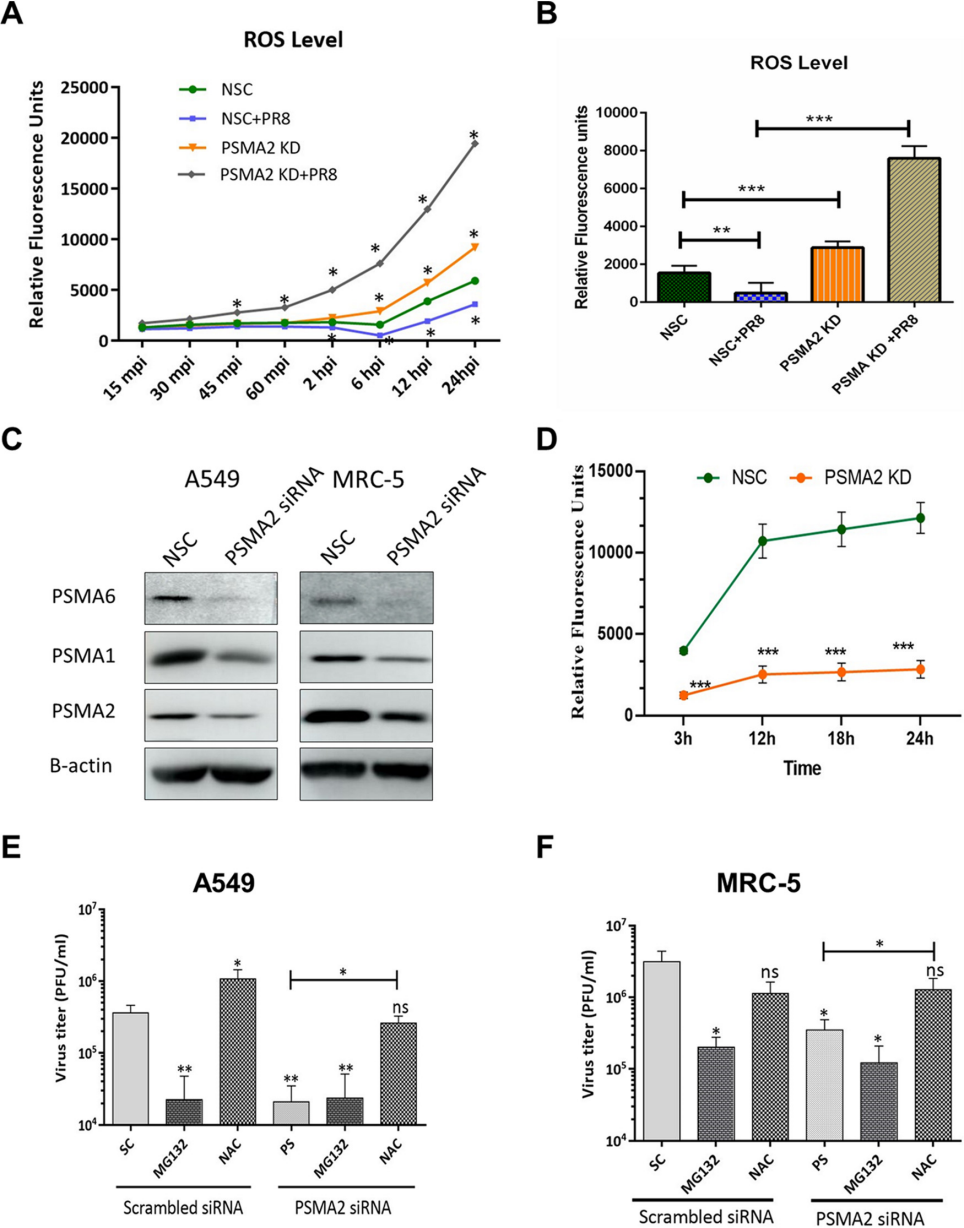
PSMA2 KD reduces proteasome activity but does not affect IAV replication in the presence of NAC. (A and B) Change in reactive oxygen species (ROS) levels over time (A) and at 6 hpi (B) by PR8 infection, PSMA2 KD, and PR8 infection in A549 cells. (C) Expression of PSMA1 and PSMA6 in A549 and MRC-5 cells after PSMA2 knockdown. (D) 20S proteasome activity in PSMA2 KD cells. (E) Impact of MG132 and NAC on IAV replication in wild-type cells and after PSMA2 KD in A549 cells. (F) Impact of MG132 and NAC on IAV replication in wild-type MRC-5 cells and after PSMA2 KD. All significance levels were calculated in comparison with NSC/SC, without the bars compared with the horizontal lines. *, *P* < 0.05; **, *P* < 0.01; ***, *P* < 0.001.

Next, we examined the impact of PSMA2 KD on reactive oxygen species (ROS) levels in IAV-infected PSMA2 KD and control cells. IAV infection caused a significant decrease in ROS level, whereas PSMA2 KD caused significant upregulation of ROS levels. Interestingly, the ROS level increases were even higher in PSMA2 KD cells during IAV infection (Fig. 6A and B). To investigate the importance of PSMA2 in the proteosome, we determined the impact of PSMA2 KD on the levels of PSMA1 and PSMA6 and on 20S proteasome activity. PSMA2 KD negatively impacted the expression of PSMA1 and PSMA6 (Fig. 6C) and significantly reduced 20S proteasome activity (Fig. 6D).

To further understand the role of PSMA2 in the NRF2-mediated oxidative response pathway, we investigated the role of MG132, a proteasome inhibitor, and *N*-acetyl-l-cysteine (NAC), an ROS scavenger, during IAV replication. MG132 caused significant inhibition of IAV in both A549 and MRC-5 cells. In contrast, NAC caused significant enhancement of IAV replication in wild-type and PSMA2 KD A549 cells (Fig. 6E and F). Interestingly, we did not observe any increase in IAV replication in wild-type MRC-5 cells after NAC treatment but IAV replication was enhanced in PSMA2 KD cells (Fig. 6F).

**FIG 7:**
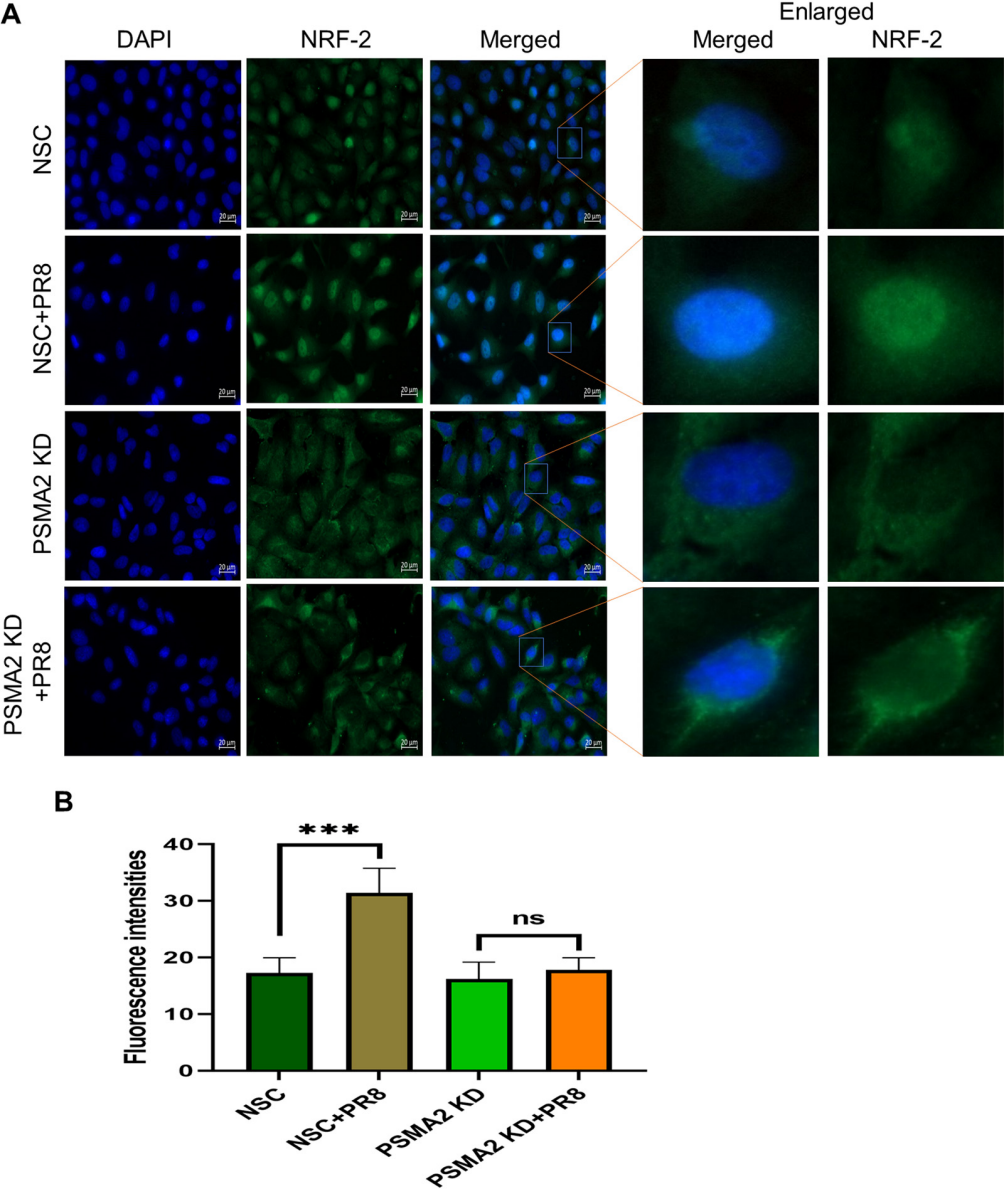
PSMA2 is required for nuclear translocation of NRF2. (A) Immunofluorescence images showing the impact of PSMA2 KD on NRF2 nuclear translocation in influenza A virus-infected A549 cells. (B) Quantitative fluorescence intensity of NRF2 in the nucleus determined by ImageJ. The nonsilencing scrambled siRNA control shows the distribution of NRF2 in noninfected cells treated with nontargeted siRNAs. NSC+PR8 indicates the nuclear translocation of NRF2 in IAV-infected cells. PSMA2 KD shows the distribution of NRF2 in noninfected, PSMA2-depleted cells. PSMA2 KD+PR8 shows the impact of PSMA2 KD on translocation of NRF2 in IAV-infected cells.

NRF2 nuclear translocation is a critical step in the NRF2-mediated oxidative response pathway. Immunofluorescence microscopy showed that IAV infection caused substantial translocation of the NRF2 proteins into the nucleus. However, during IAV infection in PSMA2 KD cells, NRF2 nuclear translocation was not significant and the protein accumulated to a larger amount in the cytoplasm (Fig. 7).

## DISCUSSION

### PSMA2 knockdown alters IAV-mediated host proteomic responses.

By proteomic analysis, we found that levels of transforming growth factor beta-induced (TGFBI), Fas cell surface death receptor (FAS), plasminogen activator urokinase (PLAU), and cathepsin B (CTSB) proteins were significantly higher in PR8-infected cells but were slightly lower in PSMA2 KD cells during IAV infection. Higher protein levels could result from protein upregulation or slower protein turnover or a combination. Conversely, proteins whose levels are lower, because of either downregulation, faster turnover, or both, are referred to as downregulated. TGFBI is involved in cell movement and transformation, but its role in viral replication is not well understood (40). In contrast, FAS (41, 42), PLAU (43), and CTSB (44) were previously identified as critical factors for IAV replication. PSMA2 KD may hinder the utilization of these proteins by IAV. However, lipocalin 2 (LCN2), cyclin-dependent kinase 2 (CDK2), and cyclin A2 (CCNA2) were significantly upregulated, and tissue factor pathway inhibitor (TFPI), growth factor receptor-bound protein 2 (GRB2), and cyclin-dependent kinase inhibitor 1B (CDKN1B) were significantly downregulated in PSMA2 KD+PR8 infection but oppositely regulated and not significant in non-KD PR8-infected cells. LCN2 is a key regulator of inflammation during mycobacterial infection (45) and deactivates macrophages (46) during viral infection. CDK2 and CCNA2 play vital roles in regulating the eukaryotic cell division cycle (47, 48), but IAV tries to arrest the cell cycle during infection (49). GRB2 and CDKN1B are also involved in regulating cell proliferation and cell cycle control (50, 51). Thus, PSMA2 KD may affect IAV replication by influencing inflammation and cell cycle regulation during infection.

IPA also showed that phosphorylation of l-amino acid and l-tyrosine was significantly downregulated by PR8 infection but not affected during PSMA2 KD+PR8 infection (Fig. 4E). Regulation of viral protein phosphorylation is critical for viral replication (52–55) and activation of cellular signaling pathways (56). However, differentiation of immune cells, like macrophages, leukocytes, and antigen-presenting cells, was downregulated by PSMA2 KD. In addition, canonical pathway analysis showed that formyl-methionine-leucyl-phenylalanine (FMLP) signaling in neutrophils, protein kinase C (PKC) signaling in T lymphocytes and FCγ RIIB signaling in B lymphocytes were downregulated by PR8 infection but upregulated by PSMA2 KD, resulting in no impact during PSMA2 KD+PR8 infection (Fig. 4C). One limitation of this study is that it was performed in an *in vitro* setting using a transformed lung epithelial cell line. Further investigation is necessary to understand the role of PSMA2 in the regulation of immune cell differentiation and activation of signaling pathways in immune cells during IAV infection using an *in vivo* experimental model.

### PSMA2 promotes IAV maturation.

PSMA2 is one of the critical alpha subunits of the 20S proteasome that build the substrate entrance gate. The 20S proteasome is an essential component of the 26S proteasome. Both of these proteasomes are pivotal components in the ubiquitin-proteasome system (UPS) and are mainly involved in cellular proteolytic modification and recycling of defective proteins (57). During a viral infection, the UPS works as a double-edged sword. Many viruses exploit the UPS to complete viral replication, whereas host cells may use it to eliminate the virus (58). Proteasomal activity is important for replication of different viruses, including entry of herpes simplex virus (59), budding of rhabdoviruses (60), DNA replication of vaccinia virus (61), genome replication of West Nile virus (27), and RNA replication and protein synthesis of coxsackievirus (62). A previous study showed that protease inhibitors can affect the entry of the IAV WSN strain (63). Although PSMA2 knockdown caused a significant reduction in proteasomal activity, it did not affect IAV entry in our study.

**FIG 8:**
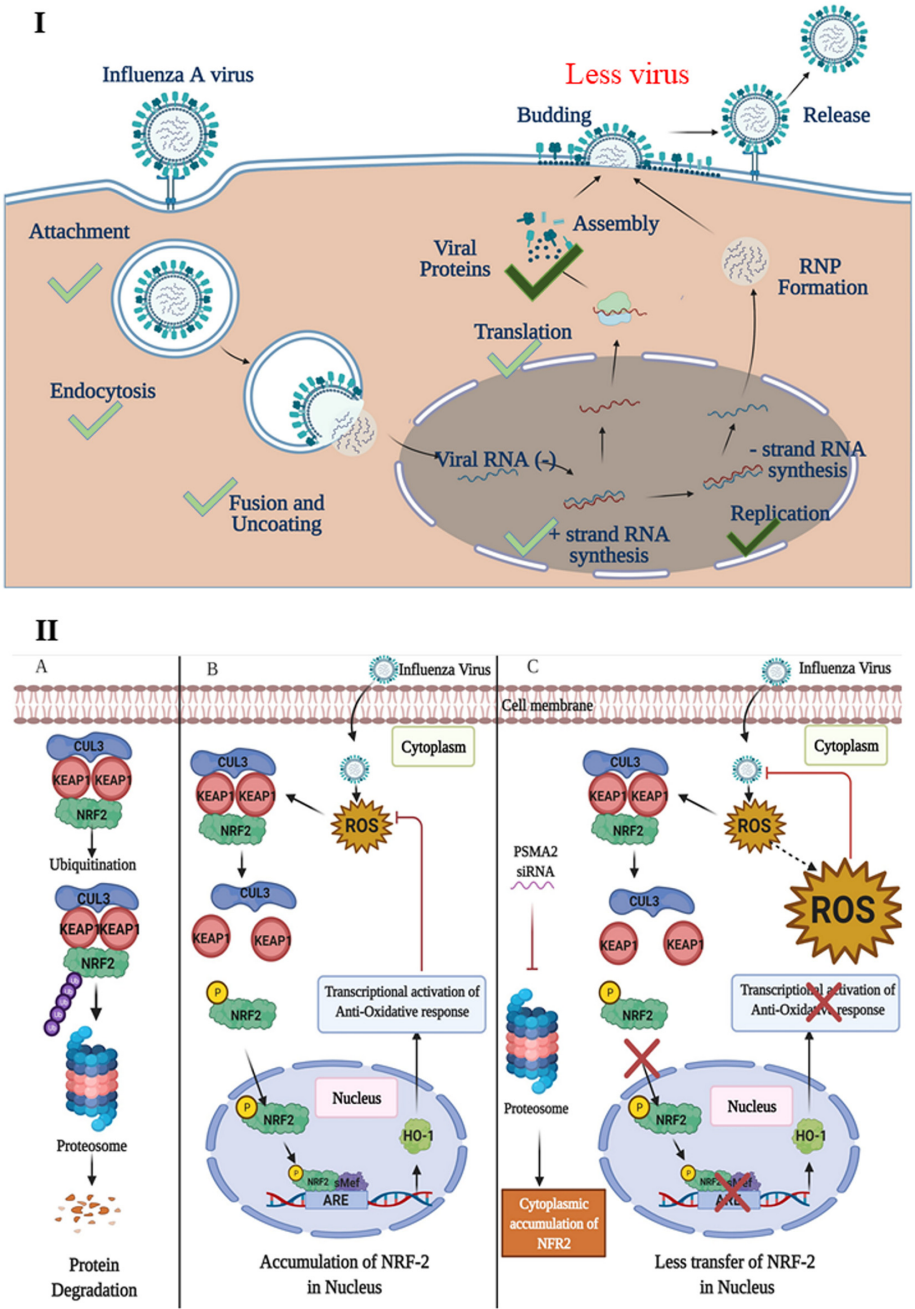
Proposed model showing the role of PSMA2 in IAV replication cycle and NRF2-mediated oxidative response pathway during IAV infection in human lung epithelial cells. (I) The translation of viral proteins and transcription of vRNAs were not affected in PSMA2 KD cells during IAV infection. This indicates that earlier steps in the IAV replication cycle (i.e., attachment, entry, nuclear transport, and mRNA synthesis) were unaffected. Significantly fewer virus progeny were detected in the supernatant of PSMA2 KD cells compared to the control. Furthermore, although viral protein expression was not affected by PSMA2 KD, higher intracellular intensities of NP proteins suggest that PSMA2 is involved in a maturation step of IAV replication. (II) (A) Normally in cells, NRF2 is located in the cytoplasm in a complex form bound with KEAP1 and CUL3. NRF2-KEAP1 complex gets recycled by ubiquitination and frequent degradation by the proteasome. (B) Viral infection and any other stress condition cause increase of the cellular ROS level. The ROS induce the NRF2-KEAP1 complex to dissociate. Then NRF-2 gets phosphorylated and translocates into the nucleus. In the nucleus, it works as a transcription activator and activates expression of antioxidant proteins. The antioxidant proteins translocate to the cytoplasm and reduce ROS levels to protect the cell from ROS-mediated cell injury. (C) IAV infection of PSMA2 KD cells causes an increase in ROS levels and subsequent dissociation of the NRF2-KEAP1 complex. But PSMA2 KD causes a significant reduction in 20S proteasome activity. Inactivation of proteasome activity may cause NRF2 accumulation in the cytoplasm and is required for nuclear translocation of the protein. Thus, the transcriptional activation of antioxidant response may not be activated, which may result in a higher level of ROS accumulation in the cells. The ROS may act on the virus and inactivate it.

PSMA2 KD caused a significant reduction of progeny supernatant infectious virus compared to the non-KD control. The translation of viral proteins and transcription of vRNAs did not appear to be affected by PSMA2 KD (Fig. 3). This indicates that earlier steps in the IAV replication cycle (e.g., attachment, entry, nuclear transport, and mRNA synthesis) were also unaffected. Although viral protein expression was not affected by PSMA2 KD, we observed higher NP protein intensities inside the PSMA2 KD cells (Fig. 3H). In summary, these data suggest that PSMA2 is involved a maturation step(s) during IAV replication (Fig. 8I). Since PSMA2, along with the proteasome, is involved in processing and modification of cellular proteins, IAV may be usurping the proteasome for viral protein processing, which is necessary for virus particle assembly or release.

Some previous studies have shown that expression of PSMA2 was upregulated in A549 cells after infection with highly pathogenic IAV strains (7) or in primary bronchial airway epithelial cells by PR8 infection (64). However, in this study, we detected a significantly lower expression of PSMA2 in A549 cells after PR8 infection (Table 2). PR8 is a lab-adapted IAV strain and may have a different replication mechanism than highly pathogenic IAV strains in the A549 cells, and it may also have a dissimilar reaction in different cells. Another study by Shahiduzzaman et al. (24) reported that IAV infection enhanced the activity profile of PSMA2. However, knockdown of PSMA2 can cause a significant reduction in 20S proteasome activity (Fig. 6C). Thus, it appears that proteasomal activity is critical for IAV assembly or maturation.

### PSMA2 knockdown affects NRF2-mediated oxidative stress.

Nuclear factor erythroid 2p45-related factor 2 (NRF2) is an antioxidative transcription factor. Under normal conditions, it is bound with Kelch-like ECH-associated protein 1 (KEAP1) and cullin-3-based E3 ubiquitin ligase (CUL3) (65). The NRF2/KEAP1 protein complex gets ubiquitinated frequently, is degraded by the proteasome (66), and turns off the activation of the NRF2-mediated oxidative response (Fig. 8IIA).

A wide range of virus infections can induce strong oxidative stress (67–69), and many of them can activate the NRF2 pathway (70, 71). Some viruses can inactivate the pathway (72–74). Interestingly, some viruses induce oxidative stress to facilitate their replication (75–78). However, oxidative stress during infection can activate antiviral signaling pathways (79, 80), an arm of innate immunity to inactivate the pathogen (81–83). On the other hand, a high level of reactive oxygen species (ROS) can damage the cell (84). Thus, in response to ROS, the cell activates the NRF2-mediated signaling pathway, which activates the transcription of antioxidative molecules for its own protection (71, 85). Therefore, an appropriate balance of oxidative response is critical for the successful completion of viral replication, preservation of cell damage, or killing of the pathogen (86–88).

IAV can induce oxidative stress, and high levels of ROS act on the NRF2-KEAP1 complex to activate the NRF2-mediated oxidative response pathway (71). NRF2 translocates to the nucleus and forms a complex with Maf and other coactivator proteins. The complex binds to the promoter of antioxidant response elements (AREs) and activates the transcription of antioxidant and cytoprotective proteins such as oxygenase-1 (HO-1), catalase (CAT), and superoxide dismutase (SOD) (85) (Fig. 8IIB). The antioxidant proteins translocate to the cytoplasm and reduce the ROS level to protect the cell from ROS-mediated cell injury (85). Interestingly, IPA predicted that PR8 infection significantly inactivated the NRF2-mediated oxidative response pathway (Fig. 5B1), but the proteomic data were collected at 24 hpi. The oxidative response pathway activates just after virus entry, and by 24 hpi, the ROS level may have already been reduced by the expression of antioxidative molecules. However, a significant reduction of ROS levels and NRF2 nuclear translocation indicates that the NRF2-mediated oxidative response pathway was activated by the IAV infection.

IAV infection of PSMA2 KD cells will cause an increase in ROS level and subsequent dissociation of the NRF2-KEAP1 complex. However, PSMA2 KD caused a significant reduction in 20S proteasome activity. Inactivation of proteasome activity may have induced an accumulation of NRF2 in the cytoplasm. Thus, it could not activate the transcription of AREs, which may result in the accumulation of a higher level of intracellular ROS. However, treatment with an ROS scavenger could reverse the impact of PSMA2 KD on IAV replication (Fig. 6E and F). These observations clearly indicate that the accumulated ROS can inactivate the virus directly or by activation of antiviral responses (Fig. 8IIC). IPA could not predict any significant activation of NRF2-mediated oxidative response pathway by IAV infection in PSMA2 KD cells (Fig. 5B3), but only PSMA2 KD caused significant activation of the pathway (Fig. 5B2). By IF microscopy, we observed that NRF2 nuclear translocation was affected by PSMA2 KD. Therefore, PSMA2 or proteasomal activity is necessary for nuclear translocation of NRF2 and activation of the pathway, but the mechanism is still not clearly understood. PSMA2 KD caused higher ROS accumulation in the cells (Fig. 6A and B), which may have pushed the pathway toward activation and was reflected in proteomic changes detected by SomaScan.

ROS play a critical role during viral pathogenesis, as it inactivates the virus by direct killing or can induce antiviral responses. IAV requires the help of PSMA2 to activate the NRF2-mediated oxidative response to escape ROS-mediated virus inactivation. Thus, we need to have a clear understanding of the role of PSMA2 in balancing the ROS-mediated antiviral response, which may aid in the development of an effective antiviral drug to combat IAV.

In conclusion, we identified PSMA2 as a critical host dependency factor for IAV replication. PSMA2 is a critical component of the proteasome. Proteasome inhibitors have been suggested as an antiviral therapy against COVID-19 and HIV (89–91). However, thousands of proteins and many cellular functions are directly connected with this protein. In the future, an antiviral drug targeting PSMA2 or proteasome activity could be developed against IAV, but this requires extensive further study to understand the mechanism more clearly. A clear understanding of the mechanism may help us to design an antiviral drug targeting a particular interaction specifically required by IAV to avoid potential off-target effects of such drugs.

## MATERIALS AND METHODS

### Cells and viruses.

Human A549 lung epithelial cells were cultured in complete Dulbecco modified Eagle medium (DMEM; Gibco, USA) containing nonessential amino acids (NEAA), sodium pyruvate, and l‐glutamine, and supplemented with 10% fetal bovine serum (FBS; Thermo Fisher Scientific, ON, Canada) following the protocol described previously (10). Human fetal lung MRC-5 cells were purchased from ATCC (catalog no. CCL-171) and maintained in ATCC Eagle minimum essential medium (EMEM; catalog no. 30-2003) supplemented with 10% FBS. All cells were incubated at 37°C in 5% CO_2_ and passaged three times each week to maintain them as monolayers. Human influenza virus strains A/Mexico/INDRE4487/2009 (H1N1; pdm09) and A/WSN/1933 (H1N1; WSN) and mouse-adapted strain A/PR/8/34 (H1N1; PR8) were used in this study. MDCK cells were infected at an MOI of 0.01 (PFU/cell), and viruses were harvested from the supernatant after 48 h. The virus stocks were concentrated by centrifugation at 64,000 × *g* for 2 h at 4°C and resuspended in phosphate-buffered saline (PBS) supplemented with 10% glycerol, and aliquots were frozen at −80°C until used.

### Infection and plaque assay.

Human A549 cells were grown to 70 to 80% confluency, washed with 1× PBS two times, and infected with PR8, WSN, or pdm09 virus. To determine the impact of PSMA2 KD on viral replication, cells were infected at an MOI of 0.01. Supernatants from the virus-infected cells were collected at 0, 2, 4, 8,16, 24, 36, and 45 h postinfection (hpi). Plaque assay was done to determine the supernatant virus titers. Samples were serially 1:10 diluted in gel saline, and 100 μl of each diluted sample was inoculated in duplicate into separate wells of 6-well plates containing monolayers of MDCK cells. The infected plates were rocked for 1 h to allow virus attachment and then overlaid with FBS-free 1× DMEM containing 0.8% Avicel, 2 mM sodium pyruvate, 2 mM l-glutamine, and 1× MEM nonessential amino acids. The overlay medium was also supplemented with antibiotics (gentamicin and amphotericin B) and 2.5 μg/mL of trypsin. The infected cells were incubated for 72 h at 35°C. After that, the overlay medium was removed, and cells were washed once with 1× PBS and fixed with 2% formaldehyde for 30 min. The plaques were visualized by staining with crystal violet for ≥1 h. The crystal violet stain was washed, plates were dried, and plaques were counted. The number of plaques was back-calculated to quantify PFU per milliliter (10).

### Cell viability.

The impact of PSMA2 KD on cell viability was determined with WST-1 (Roche) reagent according to the manufacturer’s instructions. Eight thousand A549 cells were seeded in each well of 96-well plates. After overnight incubation, cells were transfected with PSMA2 or nonsilencing scrambled (NSC) siRNAs. At 48 and 72 h posttransfection (hpt), 9 μL of WST-1 reagent was added to each well and incubated at 37°C for 2 h. Cell viability was calculated from the colorimetric changes in the media determined by a photodensitometer. The percentage of cell viability was determined by comparing with time-matched nonsilencing (NSC) cells. Each experiment was done in 3 biological replicates with 5 technical replicates each time.

### siRNA array screens.

siRNAs corresponding to scrambled control (nonsilencing) and to a subset of genes predicted to interact with the extracellular matrix protein FN-1 were purchased from Dharmacon in a 96-well format (Dharmacon, Inc.). The siRNAs were solubilized in sets of two 96-well plates, and ∼5,000 A549 cells were added to each well to knock down the corresponding gene according to the manufacturer’s directions. Cells were incubated at 37°C, and at 48 hpt, cell viability in one set of the 96-well plates was determined by WST-1 assay as described earlier. The A549 cells in the other 96-well plates were washed, infected with PR8 at an MOI of 0.02, overlaid with DMEM lacking FBS but supplemented with 2.5 μg/mL of trypsin, and incubated at 37°C for 42 h. At 42 hpi, half of the supernatant from each well was removed for virus titration. Then fresh DMEM and WST-1 were added to determine cell viability after both transfection and infection. Transfection and infection experiments were performed in three replicates.

### siRNA transfection.

Knockdown of PSMA2 protein expression was done by siRNA following the protocol described previously (92). In summary, A549 cells were grown to 30 to 40% confluency in complete DMEM with 10% FBS, washed twice before transfection with RNase-free PBS, and overlaid with complete DMEM with 10% FBS. Smart‐pool (SP) siRNAs for PSMA2 (25 nM), NSC siRNA (Dharmacon) as a control, and DharmaFECT (catalog no. T‐2001; GE Healthcare Dharmacon, Lafayette, CO) were diluted in Opti‐MEM following the manufacturer’s instructions. The diluted siRNA and DharmaFECT were combined, incubated at room temperature for 20 min, and added directly into the A549 cell culture media. Culture dishes were incubated at 37°C in 5% CO_2_. Cells were infected with virus at 48 hpt to investigate the impact of PSMA2 KD on viral protein and RNA production and progeny infectious virus.

### Protein extraction and quantification.

A549 cells in 6-well plates or in 6-cm dishes were transfected with siRNAs and infected with IAV at an MOI of 3 PFU/cell. Infections were harvested at different time points by scraping from the culture plates. After three washings in ice-cold PBS, cells were lysed by sonication in 60 μL of mammalian protein extraction reagent (M-PER, catalog no. 78501; Thermo Fisher Scientific) detergent, supplemented with 1× HALT protease inhibitor solution. The cell lysates were centrifuged at 14,000 × *g* for 10 min at 4°C to remove the insoluble cellular components. The protein concentrations in the clear supernatants were determined by the bicinchoninic acid (BCA) protein assay (Pierce; Rockford, IL) and quantified using bovine serum albumin standards.

### Immunoblotting.

Expression of viral and host cellular proteins was detected by Western blotting according to the procedure described previously (10). Equal amounts (10 to 30 μg) of protein samples were separated using 10 to 12% SDS-PAGE gels and transferred to 0.2-μm nitrocellulose membranes. Anti-PSMA1 (Invitrogen; catalog no. PA1-963), anti-PSMA2 (Cell Signaling; catalog no. 2455), anti-PSMA6 (Invitrogen; catalog no. PA576058), anti-beta-actin (Cell Signaling; catalog no. 3700S), anti-STAT3 (Cell Signaling; catalog no. 9139S), anti-CST3 (Cell Signaling; catalog no. 4280), and in-house-prepared IAV mouse-anti-NS1 and mouse-anti-NP (93) were used to detect specific proteins. Appropriate secondary horseradish peroxidase (HRP)-conjugated horse anti-mouse or anti-rabbit (Cell Signaling; catalog no. 7076 and 7074, respectively) were used to detect immune complexes. Protein bands were developed with ECL reagents and captured by Alpha Innotech FluorChemQ MultiImage III. The differences in protein expression were determined by measuring band intensities with ImageJ 1.50i (NIH, USA). The data were analyzed and graphically presented by GraphPad Prism v 9.1.0 software.

### Immunofluorescence microscopy.

A549 cells were grown on spotted slides in complete DMEM with 10% FBS at 37°C for 24 h. Then the cells were treated with 25 nM PSMA2 or NSC siRNAs for 48 h and infected with IAV PR8 at an MOI of 3. At 3 days postinfection, each spot was washed five times with 1× PBS and fixed with 4% paraformaldehyde for 15 min, then washed five times with 1× PBS, and permeabilized with 0.1% Triton X-100 for 5 min. The fixed cells were blocked overnight at 4°C by 3% bovine serum albumin (BSA). Cells were then incubated overnight with primary anti‐PSMA2 (catalog no. 2455; Cell Signaling) or anti-NRF2 antibody (Cell Signaling; catalog no. 12721S) in 3% BSA at 4°C. After that, cells were washed five times with 1× PBS and 0.2% Tween 20 (PBT) and incubated with Alexa Fluor 488-tagged anti-rabbit secondary antibody for 60 min. Finally, each spot on the slide was covered with 4′,6-diamidino-2-phenylindole (DAPI) mounting dye. The fluorescent images were visualized with a Zeiss Axio Observer Z1 inverted fluorescence microscope. Image 1.53e (NIH, USA) was used for measuring the average fluorescence intensities of NRF2 in the nucleus. Data were analyzed, and the graphs were prepared by GraphPad Prism v 9.1.0.

### SomaScan analyses.

To understand the impact of PSMA2 KD on the cellular proteome during IAV infection, cell lysates were collected from NSC, NSC+PR8, PSMA2 KD, and PSMA2 KD+PR8 cells at 24 hpi and analyzed by the SomaScan version 1.3K platform, which can simultaneously measure 1,307 proteins in up to 92 samples. During the SomaScan assay, each biologic sample was mixed with SomaLogic’s proprietary SOMAmers. Each of the SOMAmers can selectively recognize and bind to a specific human protein (39, 94). After mixing and binding of each sample in each well in 96-well plates., the SOMAmers are washed, released, hybridized to DNA microarrays, and quantified (94, 95). The expression values are generated as relative fluorescent units (RFU) which are directly proportional to the amounts of target proteins in the initial samples, as confirmed by a standard curve generated for each protein-SOMAmer pair. RFU were log_2_ converted and analyzed as described previously (96).

### ROS assay.

The cellular reactive oxygen species (ROS) levels were determined by staining with 2′,7′-dichlorofluorescin diacetate (DCF-DA; catalog no. D6883; Sigma-Aldrich) following the manufacturer’s instructions. A549 cells were cultured in 96-well plates and transfected with PSMA2 siRNA or NSC for 48 h. Cells were washed once with PBS and incubated for 45 min with 10 μM DCF-DA at 37°C. After that, the cells were infected with IAV at an MOI of 3 and incubated at 37°C in the dark. Fluorescence was measured (excitation wavelength, 504 nm; capture wavelength, 529 nm) at different time points postinfection. The experiment was performed in five replicates.

### Proteasome 20S activity assay.

Proteasome activity was assessed using a proteasome 20S assay kit according to the manufacturer’s instructions (Sigma-Aldrich; MAK172). In summary, 10,000 A549 cells were seeded in each well of black clear-bottom 96-well plates. After overnight incubation at 37°C, cells were transfected with PSMA2 siRNA as described above. At 72 hpt, cells were washed twice with PBS, and 100 μL of proteasome assay loading solution was added to each well. The 96-well plates were incubated in the dark at 37°C for 12 h, and fluorescence intensity was measured using a spectrophotometer at an excitation wavelength of 490 nm and emission wavelength of 525 nm. The impact on 20S proteasome activity was determined by comparing the relative fluorescence units in PSMA2 KD cells to that in non-KD cells.

### Impact of ROS scavenger and proteasome inhibitor on IAV replication.

An ROS scavenger, *N*-acetyl-l-cysteine (NAC; catalog no. A7250), and a proteasome inhibitor, carbobenzoxy-Leu-Leu-leucinal (MG132, catalog no. M8699) were purchased from Sigma-Aldrich. A549 and MRC-5 cells were treated with different concentrations of MG132 or NAC, and the cytotoxicity was determined by WST-1 assay at 48 h posttreatment. NAC at 15 mM and MG132 at 0.05 μM were found to be nontoxic to the cells. To understand the effects of NAC and MG132, cells were infected with IAV strain PR8 at an MOI of 0.01 and NAC or MG132 was added in the overlay media. Supernatants were collected at 45 hpi, and virus titers were determined by plaque assay.

### RNA extraction and real-time PCR.

To understand the impact of PSMA2 KD on vRNA transcription, A549 cells were infected with IAV PR8 at an MOI of 3. At 24 hpi, cells were harvested and washed with cold PBS, and total cellular mRNA was extracted with an RNeasy minikit (Qiagen). cDNA was synthesized with the GoScript reverse transcription system kit (Promega) from 250 ng of purified mRNA. The qRT-PCR was performed using a Platinum SYBR green qPCR Supermix-UDG kit (Thermo Fisher). The final volume of PSC master mix was 25 μL, consisting of 12.5 μL of Platinum SYBR green qPCR Supermix (2×), 0.5 μL of ROX reference dye, 0.5 μL each of 10 μM forward and reverse primers listed below, 6 μL of H_2_O, and 5 μL (10 ng) of template cDNA. The PCR was performed in three biological replicates and two technical replicates for each sample. All PCRs were performed and analyzed on a QuantStudio 3 real-time PCR system (Applied Biosystems). The PCR cycle conditions were 50°C for 2 min, 95°C for 2 min, and 40 cycles of 95°C for 15 s and 60°C for 30 s. The threshold cycle (*C_T_*) values were normalized to the 18S rRNA control and compared to the nontargeting siRNA control. The sequences of the primers were as follows: for PR8-NS1, CTTCGCCGAGATCAGAAATC (forward [Fwd]) and TGGACCATTCCCTTGACATT (reverse [Rev]); for PR8-NP, AGAGGGTCGGTTGCTCACAA (Fwd) and TGGCTACGGCAGGTCCATA (Rev); and for PR8-HA, CATTCCGTCCATTCAATCC (Fwd) and AACCATACCATCCATCTATC (Rev).

### Statistical and bioinformatics analyses.

The SomaScan-generated proteomic data are expressed in RFU. The protein expression numbers were converted to log_2_ values. The average log_2_ difference values were converted to fold changes for each of the proteins. To quantify the *P* value from the fold changes, Z-score analysis and Student’s *t* test (2 tailed) were performed (10). The list of significantly dysregulated proteins (*P* value < 0.05) with fold change above 1.5 or below −1.5 (Table 2) were further analyzed by Ingenuity Pathway Analysis (IPA) and STRING: functional protein association networks. Z-score values of ≥1.96σ or ≤−1.96σ were considered significant. One-way analysis of variance (ANOVA) was performed to analyze the Western blot data for calculating the *P* values. A *P* value of <0.05 was considered significant. Heat maps were plotted using MORPHEUS (Broad Institute, Cambridge, MA) and Fig. 8 was designed with BioRender (https://biorender.com/) free online software.
