# Supplementary material for: Influenza A Virus Uses PSMA2 for Downregulation of the NRF2-Mediated Oxidative Stress Response
Source: J Virol. 2022 Mar 9;96(5):e01990-21. doi: 10.1128/jvi.01990-21 (PMC8906419; doi:10.1128/jvi.01990-21)
Supplement: Supplemental file 1 — Fig. S1 and Tables S1 and S2 . Download jvi.01990-21-s0001.pdf, PDF file, 1.3 MB [file jvi.01990-21-s0001.pdf]

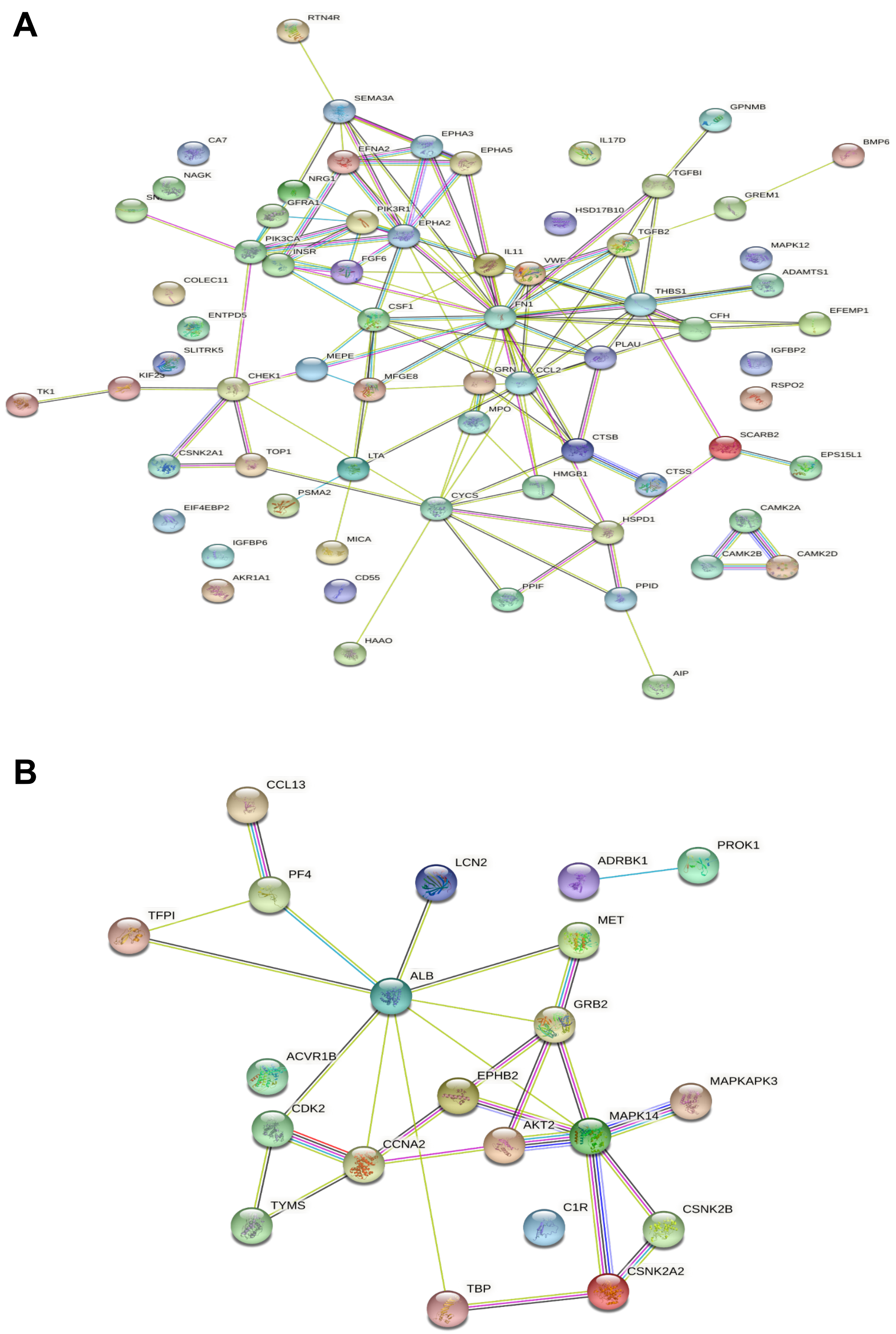

Supplementary Figure S1. **A.** Direct and indirect interactions of the proteins dysregulated in A549 cells after PR8 infected and which were not significantly affected during PR8 infection in PSMA2 KD cells. **B.** Direct and indirect interactions of the proteins dysregulated during PR8 infection in PSMA2 KD cells but were not significantly affected in A549 cells after PR8 infection.

Supplementary Table S1. Diseases and functions dysregulated by PSMA2 KD, PR8 infection, and PSMA2 KD+PR8 infection.

Diseases and Bio Functions	PSMA2 KD	PR8	PSMA2 KD +PR8
Differentiation of nervous system	-0.381	-2.814	-2.733
Phosphorylation of L-amino acid	1.038	-2.574	N/A
Differentiation of neurons	-0.404	-2.543	-2.146
Invasion of cells	1.997	-2.482	-1.312
Shock Response	N/A	-2.388	-1.475
Cell proliferation of breast cell lines	N/A	-2.359	-0.388
Proliferation of lung cancer cell lines	N/A	-2.325	-1.749
Outgrowth of neurites	1.277	-2.202	-2.16
Septic shock	N/A	-2.177	-1.186
Neovascularization of organ	N/A	-2.157	N/A
Proliferation of neuronal cells	1.631	-2.126	-2.049
Binding of blood cells	-0.135	-2.117	N/A
Adhesion of immune cells	N/A	-2.101	-0.67
Growth of malignant tumor	1.871	-2.09	-1.822
Growth of neurites	1.449	-2.065	-1.971
Cell movement	2.539	-1.99	-2.259
Phosphorylation of L-tyrosine	N/A	-1.977	N/A
Proliferation of epithelial cells	2.16	-1.933	-0.06
Cell proliferation of breast cancer cell lines	2.979	-1.885	-1.904
Cell viability of tumor cell lines	3.639	-1.784	-0.824
Cell viability	2.665	-1.763	-1.775
Cell death of cerebral cortex cells	-1.968	-1.714	-1.265
Hematopoiesis of mononuclear leukocytes	-0.457	-1.615	-2.651
Cell proliferation of tumor cell lines	2.94	-1.598	-0.831
Migration of cells	2.289	-1.563	-2.332
Sprouting	N/A	-1.552	-2.622
Leukopoiesis	0.294	-1.506	-2.41
Differentiation of mononuclear leukocytes	N/A	-1.458	-2.401
Growth of embryo	0.674	-1.415	-2.288
Development of genitourinary system	N/A	-1.37	-2.102
Tubulation of cells	2.177	-1.37	-1.455
Differentiation of phagocytes	N/A	-1.36	-2.416
Growth of epithelial tissue	2.777	-1.256	0.436
Growth of carcinoma	N/A	-1.189	-2.233
Growth of tumor	2.35	-1.009	-0.854
Advanced malignant solid tumor	2.418	-0.97	-1.732
Metastatic solid tumor	2.418	-0.97	-1.732
Visceral metastasis	2.207	-0.888	-1.192
Advanced extracranial solid tumor	2.2	-0.824	-1.067
Growth of organism	2.005	-0.733	-0.905
Cell movement of tumor cell lines	3.043	-0.731	-0.327
Colony formation of cells	2.127	-0.684	N/A
Advanced malignant tumor	3.063	-0.567	-2.332
Secondary tumor	3.063	-0.567	-2.332

Migration of tumor cell lines	2.478	-0.168	-0.228
Extracranial solid tumor	2.039	-0.054	N/A
Necrosis	-2.143	0.066	0.139
Neoplasia of cells	2.608	0.096	-1.453
Cell death of tumor cell lines	-2.43	0.146	-1.019
Migration of embryonic cell lines	2.915	0.149	-0.551
Hemostasis	1.976	0.589	1.798
Proliferation of endothelial cells	2.296	0.59	0.592
Apoptosis of tumor cell lines	-2.842	0.688	-0.738
Cell movement of fibroblast cell lines	2.923	0.72	-0.895
Proliferation of muscle cells	-0.365	0.749	2.754
Development of endothelial tissue	2.023	0.835	0.496
Apoptosis	-2.075	0.842	0.392
Activation of blood platelets	N/A	1.342	2
Coagulation	N/A	1.886	2.753
Apoptosis of carcinoma cell lines	-0.661	2.109	N/A
Bleeding	-0.782	2.139	N/A
Organismal death	-2.422	5.526	4.247
Branching of cells	1.662	N/A	-2.404
Differentiation of antigen presenting cells	N/A	N/A	-2.226
Metastatic potential	N/A	N/A	-2.219
Differentiation of myeloid leukocytes	N/A	N/A	-2.209
Differentiation of macrophages	N/A	N/A	-1.964
Differentiation of embryonic tissue	2.245	N/A	-1.414
Cell movement of embryonic cell lines	2.355	N/A	-1.117
Migration of epithelial cell lines	2.607	N/A	-0.551
Thrombus	1.969	N/A	0.86
Coagulation of blood	N/A	N/A	2.376
Formation of dendrites	-2.183	N/A	N/A
Function of neurons	1.982	N/A	N/A
Replication of RNA virus	2.024	N/A	N/A
Catabolism of protein	2.145	N/A	N/A
Differentiation of stromal cells	2.159	N/A	N/A
Cell viability of brain cells	2.183	N/A	N/A
Replication of Influenza A virus	2.407	N/A	N/A

Values are Z-scores. Diseases and functions significantly upregulated (Z-score > 1.96) are indicated in red; those significantly downregulated (Z_score < -1.96) are in blue.

Supplementary Table S2. Canonical Pathways significanly dysregulated by PSMA2 KD, PR8 infection, and PSMA2 KD+PR8 infection.

Canonical Pathway	PSMA2 KD	PR8	PSMA2 KD+ PR8
Cyclins and Cell Cycle Regulation	-2	N/A	N/A
Role of NFAT in Regulation of the Immune Response	0.447	-2.449	N/A
Opioid Signaling Pathway	0.816	-2.828	N/A
EIF2 Signaling	1	-2.449	N/A
Paxillin Signaling	1	-2.449	N/A
GDNF Family Ligand-Receptor Interactions	1	-2.236	N/A
cAMP-mediated signaling	1	-2	N/A
Synaptic Long Term Potentiation	1	-2	N/A
Dopamine-DARPP32 Feedback in cAMP Signaling	1	-2	N/A
GNRH Signaling	1.342	-2.236	N/A
G Beta Gamma Signaling	1.342	-2	N/A
Type I Diabetes Mellitus Signaling	1.342	-2	N/A
fMLP Signaling in Neutrophils	2	-2.646	N/A
NGF Signaling	2	-2.449	N/A
PKCθ Signaling in T Lymphocytes	2	-2.236	N/A
ErbB4 Signaling	2	-2.236	N/A
Regulation of eIF4 and p70S6K Signaling	2	-2	N/A
PAK Signaling	2	-2	N/A
FcγRIIB Signaling in B Lymphocytes	2	-2	N/A
PI3K Signaling in B Lymphocytes	2	-1.89	N/A
Gαi Signaling	2	N/A	N/A
Ephrin Receptor Signaling	2	N/A	N/A
Apelin Liver Signaling Pathway	2	N/A	N/A
Actin Cytoskeleton Signaling	2	N/A	N/A
Cholecystokinin/Gastrin-mediated Signaling	2.236	-2.236	N/A
Neurotrophin/TRK Signaling	2.236	N/A	N/A
Phospholipase C Signaling	2.449	-2.449	N/A
NRF2-mediated Oxidative Stress Response	2.449	-2	N/A
Fcγ Receptor-mediated Phagocytosis in Macrophages and Monocytes	N/A	-2.449	N/A
Neuropathic Pain Signaling In Dorsal Horn Neurons	N/A	-2.449	N/A
Gα12/13 Signaling	N/A	-2.449	N/A
Estrogen-Dependent Breast Cancer Signaling	N/A	-2.449	N/A
CNTF Signaling	N/A	-2.449	N/A
UVC-Induced MAPK Signaling	N/A	-2.236	N/A
Aldosterone Signaling in Epithelial Cells	N/A	-2.236	N/A
Antiproliferative Role of Somatostatin Receptor 2	N/A	-2.236	N/A
Lymphotoxin β Receptor Signaling	N/A	-2.236	N/A
Th1 Pathway	N/A	-2.236	N/A
IL-9 Signaling	N/A	-2	N/A
IL-22 Signaling	N/A	-2	N/A
Signaling by Rho Family GTPases	N/A	-2	N/A
Calcium-induced T Lymphocyte Apoptosis	N/A	-2	N/A
Melanoma Signaling	N/A	-2	N/A

Telomerase Signaling	N/A	-2	N/A
Inhibition of Angiogenesis by TSP1	N/A	-2	N/A
Relaxin Signaling	N/A	-2	N/A
UVA-Induced MAPK Signaling	N/A	-2	N/A
IL-2 Signaling	N/A	-2	N/A
Endometrial Cancer Signaling	N/A	-2	N/A
SAPK/JNK Signaling	N/A	-2	N/A
Corticotropin Releasing Hormone Signaling	N/A	-2	N/A
Non-Small Cell Lung Cancer Signaling	N/A	-2	N/A
LXR/RXR Activation	N/A	2	N/A
PTEN Signaling	-1.265	2.121	2.236
Small Cell Lung Cancer Signaling	-1	-2	-1
Glioblastoma Multiforme Signaling	0	-2.449	-1
CD28 Signaling in T Helper Cells	1.633	-2.121	-1
STAT3 Pathway	N/A	-2.333	-1
Acute Phase Response Signaling	2.121	-1.897	-1.134
B Cell Receptor Signaling	1.265	-2.111	-1.342
Growth Hormone Signaling	2	-2.121	-1.342
Gαq Signaling	2.449	-2.828	-1.342
IL-6 Signaling	0.333	-2.138	-1.508
Renin-Angiotensin Signaling	1.342	-2.53	-1.633
IL-7 Signaling Pathway	-0.378	-2.646	-1.89
HMGB1 Signaling	1	-2.496	-1.89
HGF Signaling	1.633	-2.828	-1.89
IL-3 Signaling	2.449	-2.714	-1.89
IL-8 Signaling	N/A	-2.333	-1.89
Dendritic Cell Maturation	N/A	-2.333	-1.89
Adrenomedullin signaling pathway	0.707	-2.828	-2
Role of NANOG in Mammalian Embryonic Stem Cell Pluripotency	1	-2.449	-2
CREB Signaling in Neurons	1	-2.449	-2
p70S6K Signaling	1.342	-2.646	-2
ERK/MAPK Signaling	1.633	-2.646	-2
Huntington's Disease Signaling	1.633	-2.121	-2
ErbB Signaling	1.89	-2.828	-2
RANK Signaling in Osteoclasts	2	-3	-2
Endothelin-1 Signaling	2	-2.828	-2
LPS-stimulated MAPK Signaling	2	-2.646	-2
Thrombopoietin Signaling	2	-2.449	-2
Leukocyte Extravasation Signaling	2.236	-3	-2
Prolactin Signaling	2.236	-2.449	-2
3-phosphoinositide Biosynthesis	N/A	-2.828	-2
Apelin Endothelial Signaling Pathway	N/A	-2.828	-2
mTOR Signaling	N/A	-2.828	-2
Superpathway of Inositol Phosphate Compounds	N/A	-2.828	-2
UVB-Induced MAPK Signaling	N/A	-2.828	-2
CXCR4 Signaling	N/A	-2.828	-2
CCR3 Signaling in Eosinophils	N/A	-2.646	-2
Macropinocytosis Signaling	N/A	-2.449	-2

GM-CSF Signaling	N/A	-2.449	-2
P2Y Purigenic Receptor Signaling Pathway	N/A	-2.449	-2
PEDF Signaling	N/A	-2.449	-2
p38 MAPK Signaling	N/A	-2.236	-2
Acute Myeloid Leukemia Signaling	N/A	-2.236	-2
SPINK1 General Cancer Pathway	N/A	-2.236	-2
Glioma Signaling	N/A	-2.121	-2
Renal Cell Carcinoma Signaling	N/A	-2	-2
Amyotrophic Lateral Sclerosis Signaling	N/A	-1	-2
FGF Signaling	2.236	-2.53	-2.121
14-3-3-mediated Signaling	0	-2.828	-2.236
Endocannabinoid Developing Neuron Pathway	0.816	-2.828	-2.236
Cardiac Hypertrophy Signaling	1.134	-3.162	-2.236
AMPK Signaling	1.342	-2.828	-2.236
Type II Diabetes Mellitus Signaling	1.342	-2.828	-2.236
Role of NFAT in Cardiac Hypertrophy	1.667	-3.207	-2.236
CD40 Signaling	2	-2.828	-2.236
Fc Epsilon RI Signaling	2.236	-2.828	-2.236
NF-κB Activation by Viruses	2.236	-2.449	-2.236
PDGF Signaling	2.828	-2.333	-2.236
Apelin Cardiomyocyte Signaling Pathway	N/A	-2.828	-2.236
Mouse Embryonic Stem Cell Pluripotency	N/A	-2.828	-2.236
VEGF Family Ligand-Receptor Interactions	N/A	-2.449	-2.236
Ovarian Cancer Signaling	N/A	-2.236	-2.236
FAT10 Cancer Signaling Pathway	N/A	-2.236	-2.236
Leptin Signaling in Obesity	N/A	-2.236	-2.236
IL-17A Signaling in Airway Cells	N/A	-2.121	-2.236
eNOS Signaling	1	-2.449	-2.449
NF-κB Signaling	1.414	-3.317	-2.449
Tec Kinase Signaling	1.89	-3.162	-2.449
FLT3 Signaling in Hematopoietic Progenitor Cells	2	-3	-2.449
Thrombin Signaling	2.236	-3	-2.449
IGF-1 Signaling	2.53	-2.828	-2.449
Nitric Oxide Signaling in the Cardiovascular System	N/A	-2.449	-2.449
Colorectal Cancer Metastasis Signaling	0	-3	-2.646
GP6 Signaling Pathway	0.447	-3	-2.646
Production of Nitric Oxide and Reactive Oxygen Species in Macrophages	1	-2.53	-2.646
Pancreatic Adenocarcinoma Signaling	1.342	-2.449	-2.646
EGF Signaling	2.646	-3.162	-2.646

Values are Z-scores. Diseases and functions significantly upregulated (Z-score > 1.96) are indicated in red; those significantly downregulated (Z_score < -1.96) are in blue.
